# Multifunctional Engineered Metal–Organic Frameworks as Targeted Protein Degraders for Augmenting Cancer Therapy via Hexokinase 2 Degradation and Provoking Cuproptosis

**DOI:** 10.34133/research.1217

**Published:** 2026-03-31

**Authors:** Shasha Li, Runjie Liu, Qixuan Zhang, Jiahui Sun, Hao Hao, Chenhao Yao, Li Yan, Dan Yang, Dechun Liu

**Affiliations:** ^1^ Research & Development Institute of Northwestern Polytechnical University in Shenzhen, Guangdong 518057, China.; ^2^ School of Life Science and Technology, Northwestern Polytechnical University, Xi’an, Shaanxi 710072, China.; ^3^ Department of Pharmaceutical Sciences, School of Biological and Pharmaceutical Sciences, Shaanxi University of Science and Technology, Weiyang University Park, Xi’an 710021, China.

## Abstract

Cuproptosis, a newly identified form of copper-dependent programmed cell death, has emerged as a potential therapeutic strategy for cancer treatment. However, its antitumor efficacy was strictly limited by dysregulated glycolytic metabolism of tumor cells. Herein, we proposed multifunctional copper-based nano-PROTACs (CHNDs) to degrade hexokinase 2 (HK-2) and amplify cuproptosis for cancer therapy via dual mitochondrial energy depletion. Our initial evaluation indicated that polyethyleneimine (PEI)-based nano-PROTACs (PHDs) triggered HK-2 degradation via the ubiquitin–proteasome system (UPS). PHDs reduced HK-2 expression to 43.7% in 4T1 cells and 42.1% in CT26 cells, which consequently impaired glycolysis in tumor cells. Furthermore, copper ion release from CHNDs in a controlled manner and the glucose metabolism homeostasis interference by PHDs orchestrally induced effective cuproptosis and glycolysis inhibition for suppressing development and metastasis of tumor cells. Notably, CHNDs markedly inhibited the growth of murine colon and breast tumors by simultaneously disrupting mitochondrial respiration and glycolysis, resulting in tumor inhibition rates of 76.6% and 55.3%, respectively, and extending the median survival of CT26 tumor-bearing mice from 21 to 29 d. Our designed multifunctional engineered nanoparticle-based targeted protein degraders facilitated a new paradigm for precise oncology and proteolysis-targeting chimera (PROTAC) development.

## Introduction

Copper, an essential micronutrient, is indispensable for a wide range of fundamental biological functions [1,2]. However, its influence on cellular function is characterized by a delicate balance between essentiality and toxicity. Excess intracellular copper can catalyze Fenton-like reactions, generating highly reactive oxygen species (ROS) that induce oxidative damage to proteins, lipids, and DNA [3,4]. In 2022, Tsvetkov and colleagues [5,6] identified a copper-dependent form of regulated cell death termed cuproptosis, providing new insight into copper-mediated cytotoxicity in cancer cells. Mechanistically, copper ionophores, such as elesclomol (ES) and disulfiram (DSF), introduce excess copper into cells, leading to copper accumulation in mitochondria. Copper directly binds to the acylated proteins of the tricarboxylic acid (TCA) cycle, promotes their aggregation, destabilizes iron–sulfur cluster proteins, induces proteotoxic stress, and ultimately results in cuproptosis of cells [7,8]. However, inhibition of mitochondrial respiration alone often yields limited therapeutic efficacy [9], as many tumor cells preferentially rely on aerobic glycolysis and maintain low cytoplasmic copper levels through tight copper homeostasis, thereby resisting copper-induced cell death [10–12].

Tumor cells are characterized by uncontrolled proliferation, a hallmark that necessitates a robust and efficient energy supply [13–15]. Unlike normal cells, tumor cells predominantly rely on aerobic glycolysis even under normoxic conditions, a phenomenon known as the Warburg effect [16]. This metabolic reprogramming not only supports rapid proliferation, angiogenesis, and immune evasion but also contributes to tumor tolerance toward cuproptosis [17]. Hexokinase 2 (HK-2), a rate-limiting enzyme catalyzing the first committed step of glycolysis, plays a central role in maintaining elevated glycolytic flux in tumor cells [18]. Beyond its metabolic function, HK-2 also modulates autophagy and attenuates cell death signaling, rendering it a pivotal node in tumor metabolic adaptation and survival [19–21]. Consequently, inhibition of HK-2 has been shown to suppress tumor progression and enhance the efficacy of copper-induced cell death [22,23].

Although small-molecule HK-2 inhibitors such as 2-deoxyglucose, 3-bromopyruvate, and lonidamine exhibit antitumor activity, their clinical application is severely limited by systemic toxicity, poor selectivity, and drug resistance [24]. Proteolysis-targeting chimeras (PROTACs) provide an alternative, event-driven strategy by inducing selective ubiquitination and proteasomal degradation of target proteins [25,26]. Despite encouraging clinical progress of several PROTAC candidates [27,28], their use in tumor metabolic regulation remains constrained by inefficient tumor delivery, limited bioavailability, off-target toxicity, and constitutive degradation activity [29,30].

Mitochondria-targeted nanomedicines have demonstrated considerable potential in cancer therapy. Notably, Li et al. [31] established a solid theoretical basis for mitochondria-targeted nanocarriers, while Shen et al. [32] reported a parallel nanoplatform strategy for targeting mitochondrial redox balance. Consequently, dual suppression of glycolytic and mitochondrial metabolic pathways has emerged as a promising strategy to enhance antitumor efficacy [33,34]. Meanwhile, the nonselective nature of cuproptosis underscores the urgent need for targeted delivery systems capable of selectively inducing copper-dependent cell death in tumor tissues while sparing normal cells. Recent advances in nanotechnology have enabled the development of multifunctional nanocarriers that facilitate precise copper delivery and tumor-specific accumulation [35–41]. Among these, copper-based metal–organic frameworks (Cu-MOFs) have attracted considerable attention due to their facile fabrication, high tunability, and exceptional copper-loading capacity, allowing sustained copper release and efficient induction of cuproptosis in cancer cells [42–44].

Collectively, despite substantial advances in copper-induced tumor therapy and PROTAC-based metabolic regulation, these 2 strategies have largely been investigated independently, thereby creating a critical gap in integrated therapeutic development. Existing copper-delivery systems primarily induce mitochondrial dysfunction but fail to suppress glycolytic compensation [45].

**Fig. 1.**
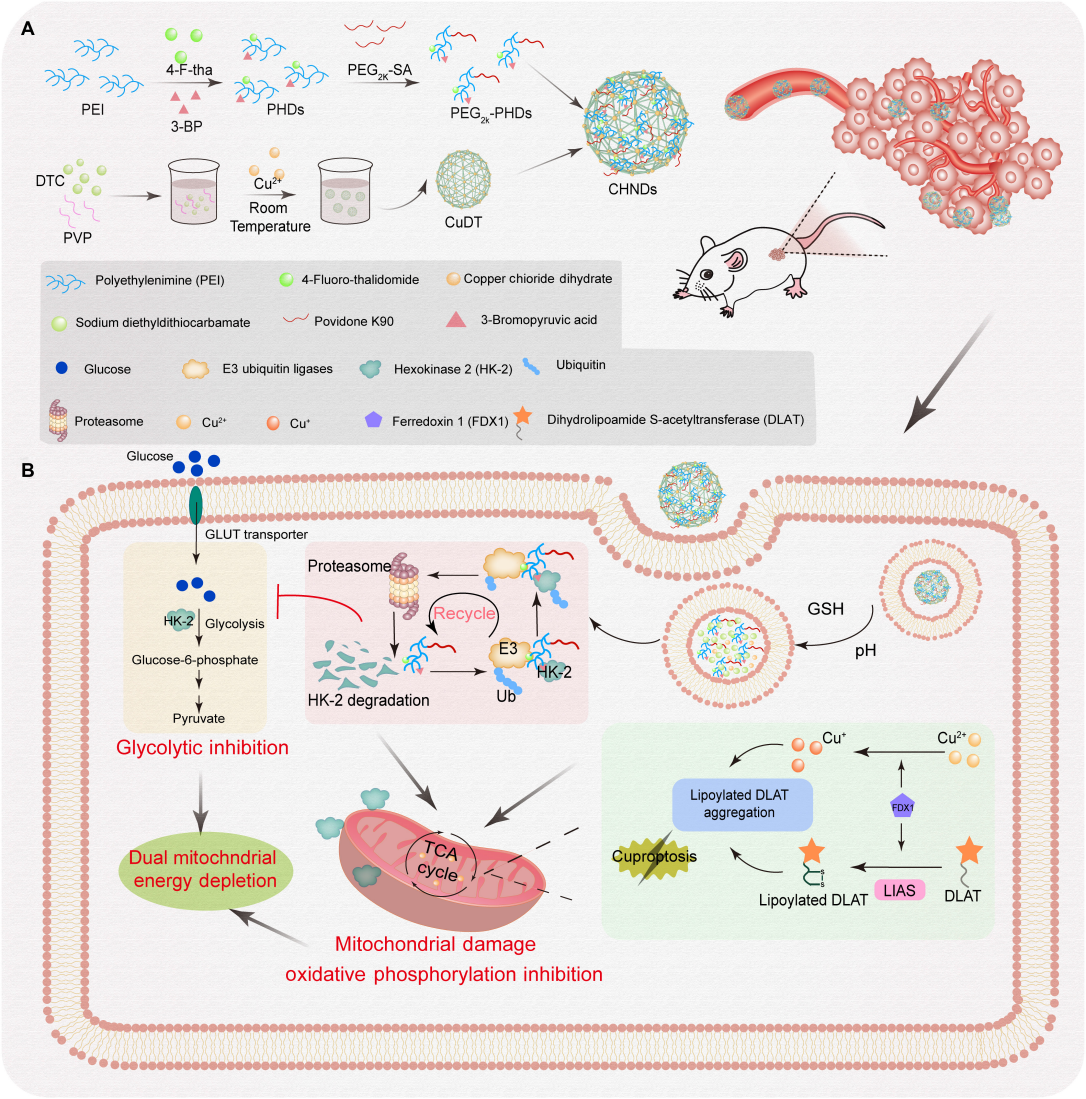
Preparation and antitumor mechanism of CHNDs. (A) Schematic diagram of the preparation of CHNDs. (B) Schematic illustration of copper-based HK-2 nano-PROTACs for the regulation of copper homeostasis and glycolysis in tumors enabling orchestrally synergistic treatment of cancers. CHNDs degraded HK-2 via the UPS pathway, achieving regulation of tumor aerobic glycolysis. Released copper ions induced lipid acylation aggregation of DLAT protein in the TCA cycle, destroyed mitochondrial function, and triggered copper death mechanism. CHNDs achieved efficient antitumor effect by simultaneously blocking glycolysis and mitochondrial respiration, and activating the cuproptosis pathway.

Herein, we developed a multifunctional copper-based HK-2 nano-PROTAC platform (CHNDs) that integrates copper-mediated cuproptosis with targeted degradation of HK-2 to overcome metabolic compensation in tumors. Following systemic administration, CHNDs preferentially accumulated in tumor tissues via the enhanced permeability and retention (EPR) effect and were internalized by cancer cells. In the tumor microenvironment, CHNDs released copper ions, leading to mitochondrial copper overload and induction of cuproptosis, while simultaneously promoting polyethylene glycol (PEG)-PHDs induced ubiquitin–proteasome system (UPS)-mediated degradation of HK-2, thereby suppressing glycolytic flux (Fig. 1). This dual-pathway intervention synergistically disrupted tumor energy metabolism and markedly inhibited tumor growth. Both in vitro and in vivo studies demonstrated that CHNDs effectively induced cuproptosis and HK-2 degradation, resulting in significant suppression of primary tumor growth and metastasis in multiple murine tumor models. Collectively, this work demonstrates a promising therapeutic strategy that combines glycolysis inhibition with copper-dependent cell death to improve cancer treatment outcomes.

## Results

### PHDs degraded HK-2 via the UPS pathway

**Fig. 2.**
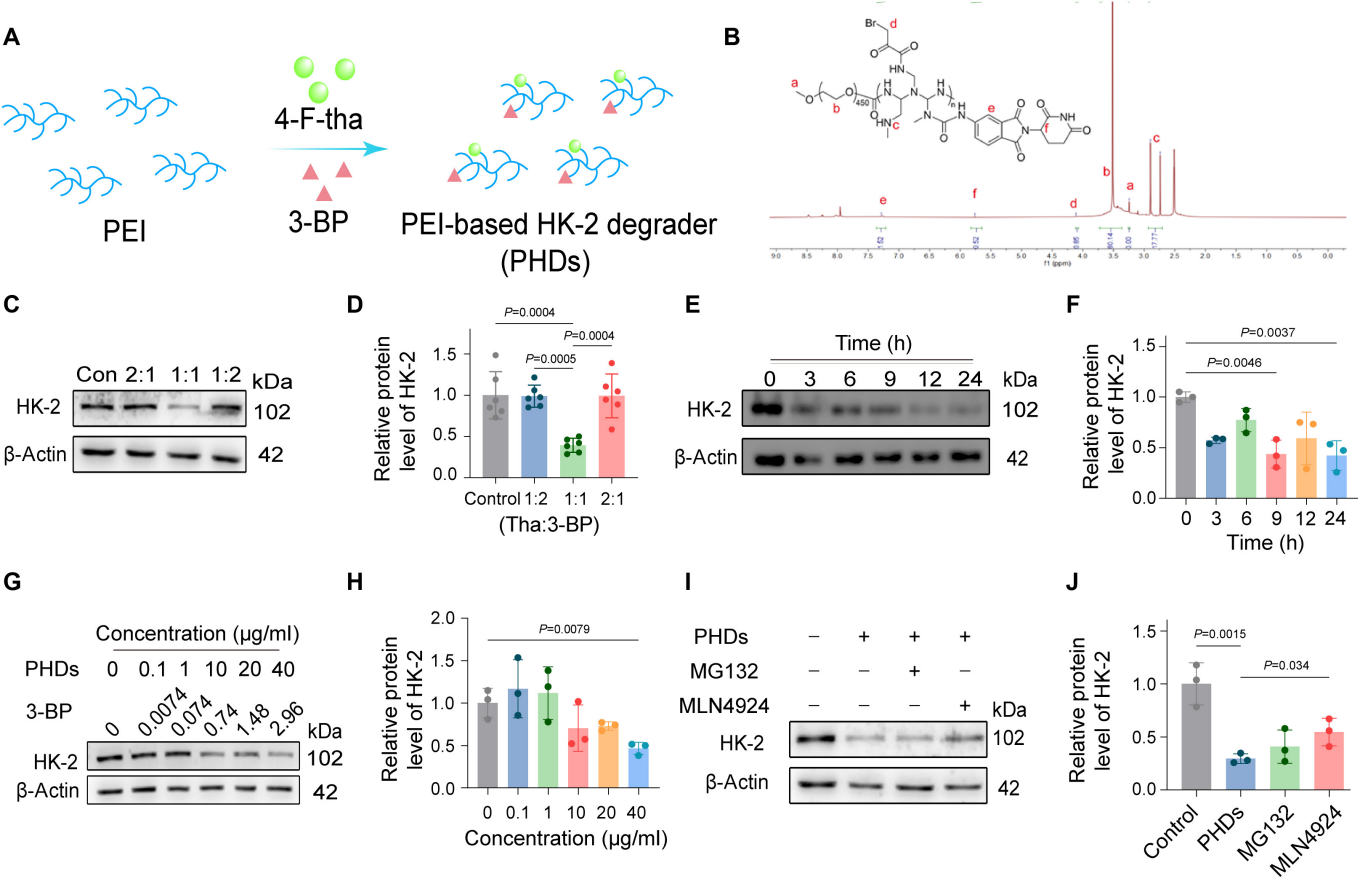
Degradation of HK-2 induced by PHDs. (A) Schematic illustration of PHD synthesis. (B) Chemical structure and ^1^H NMR spectrum of PEG-PHDs. (C) Western blot (WB) assay of PHDs with different ratios of THA and 3-BP in 4T1 cells. The ratios referred to the mass ration of PEI polymers:3-BP:THA. (D) Quantitative data of (C), and β-actin was employed as the control reference. (E) WB analysis of time-dependent HK-2 degradation in PHDs-treated 4T1 cells (0, 3, 6, 9, 12, and 24 h). (F) Quantitative data of (E), and β-actin was employed as the control reference. (G) Concentration-dependent degradation of HK-2 in 4T1 cells after 9 h of treatment with varying concentrations of PHDs. (H) Quantitative analysis of concentration-dependent HK-2 degradation induced by PHDs. (I) To investigate the mechanism of PHDs-mediated HK-2 degradation, 4T1 cells were pretreated with MG132 or MLN4924 (100 nM) for 1 h, followed by co-incubation with PHDs and either MG132 or MLN4924 for an additional 9 h. (J) Quantifying the degradation mechanism of PHDs on HK-2. All significance analyses in this figure were performed using one-way ANOVA.

Numerous tumor cells exhibit a pronounced reliance on glycolysis for energy production. This metabolic preference for glycolysis facilitates both tumor cell proliferation and immune evasion. HK-2 catalyzes the phosphorylation of glucose to glucose-6-phosphate, constituting the rate-limiting and irreversible first step of glycolysis and thereby playing a pivotal role in regulating glycolytic flux [22]. To effectively reprogram abnormal tumor glycolysis, PEI-based HK-2 degraders (PHDs) were designed and synthesized via amide bond formation between PEI and 2 distinct ligands: 4-fluorothalidomide (thalidomide derivative targeting cereblon E3 ligase) and 3-bromopyruvic acid (3-BP; a reactive warhead targeting the cysteine residues of the targeted protein HK-2) as shown in Fig. 2A. The residual amine functionalities displayed on the surface of PEI were subjected to PEGylation with methoxy PEG (mPEG_2k_) derivative for conferring CHNDs with long blood circulation and avoiding nonspecific clearance by the macrophage system in the body. The obtained PEG-PHD conjugate was subsequently purified via precipitation in ice-cold diethyl ether, subsequently coupled with extensive dialysis against deionized water. Successful conjugation was corroborated using ^1^H nuclear magnetic resonance (NMR), with the spectral data provided in Fig. 2B. Specifically, the appearance of characteristic PEG methylene proton resonances served as a definitive confirmation of mPEG_2k_ conjugation to PEI polymers. Fourier transform infrared (FTIR) spectroscopy results were depicted in Fig. S1. In the FTIR spectrum of PHDs, the N–H stretching vibration peak of the free PEI amine groups was observed at 3,450 cm^−1^, while the amide I band appeared at 1,680 cm^−1^. The absence of a fluorobenzene-specific stretching vibration peak demonstrated successful conjugation. The amide I band was also observed at 1,680 cm^−1^ in the PEG-PHDs spectrum. Additionally, the characteristic O–H stretching vibration peak of PEG appeared at 3,650 cm^−1^. These results confirmed the successful conjugation of 3-BP and 4-fluoro-thalidomide (4-F-THA) to PEI, yielding PHDs and subsequent PEGylation to obtain PEG-PHDs. This modular design allowed for the fabrication of bifunctional PEI-based PROTACs with the capacity to concomitantly engage the targeted protein and the E3 ligase, thereby facilitating the degradation of protein of interest (POI). The ratio of HK-2 and CRBN targeting warhead was calibrated through HK-2 degradation efficiency. We found that the ratio of 3-BP:THA was 1:1, achieving HK-2 degradation to 39.2 ± 7.7% (*P* = 0.005). While the ratio of 3-BP:THA was 1:2 or 2:1, the protein level of HK-2 was not affected significantly upon PHDs cocultured with 4T1 cells for 9 h (Fig. 2C and D). Then, this ratio (PEI:3-BP:THA = 1:1:1) was adopted for subsequent experiments.

The degradation level of HK-2 in 4T1 cells was positive correlation with PHDs incubation time and achieved maximum at 24 h verified by the Western blot (WB) assay (Fig. 2E and F). To avoid nonspecific protein degradation caused by cytotoxicity induced by prolonged drug incubation, this study selected 9 h as the incubation time for the concentration-dependent experiment. WB assay demonstrated that PHDs induced concentration-dependent degradation of HK-2 in 4T1 cells (Fig. 2G and H). HK-2 degradation induced by PHDs was also verified in CT26 cells, which was in accordance with 4T1 cells (Figs. S2 and S3). It was worth noting that PHDs induced HK-2 degradation with a maximal percentage degradation to 43.7 ± 11.3% in 4T1 cells and 42.1 ± 7.3% in CT26 cells. In addition, we wondered whether PHDs provoked HK-2 degradation by the UPS system as we expected or not. Subsequently, thalidomide (competitive antagonist of E3 ubiquitin ligase), MG132 or MLN4924 (proteasome inhibitors), and PHDs were co-incubated with 4T1 cells for a duration of 9 h. 4T1 cells cocultured with PHDs in the presence of inhibitors showed that the effect of degradation of HK-2 was reversed (Fig. 2I and J). Compared with the group treated with PHDs alone, MG132 and MLN4924 pretreatment restored the protein level of HK-2 from 29.4% to 40.8% and 54.6%, respectively. The HK-2 degradation mechanism induced by PHDs was also ascertained in CT26 cells. As demonstrated by the results, HK-2 protein expression in CT26 cells was partially restored following cotreatment with the proteasome inhibitor and PHDs, consistent with the previous findings (Fig. S4). These results confirmed that PHDs potently promoted HK-2 degradation through the UPS.

### Preparation and characterization of CHNDs

**Fig. 3.**
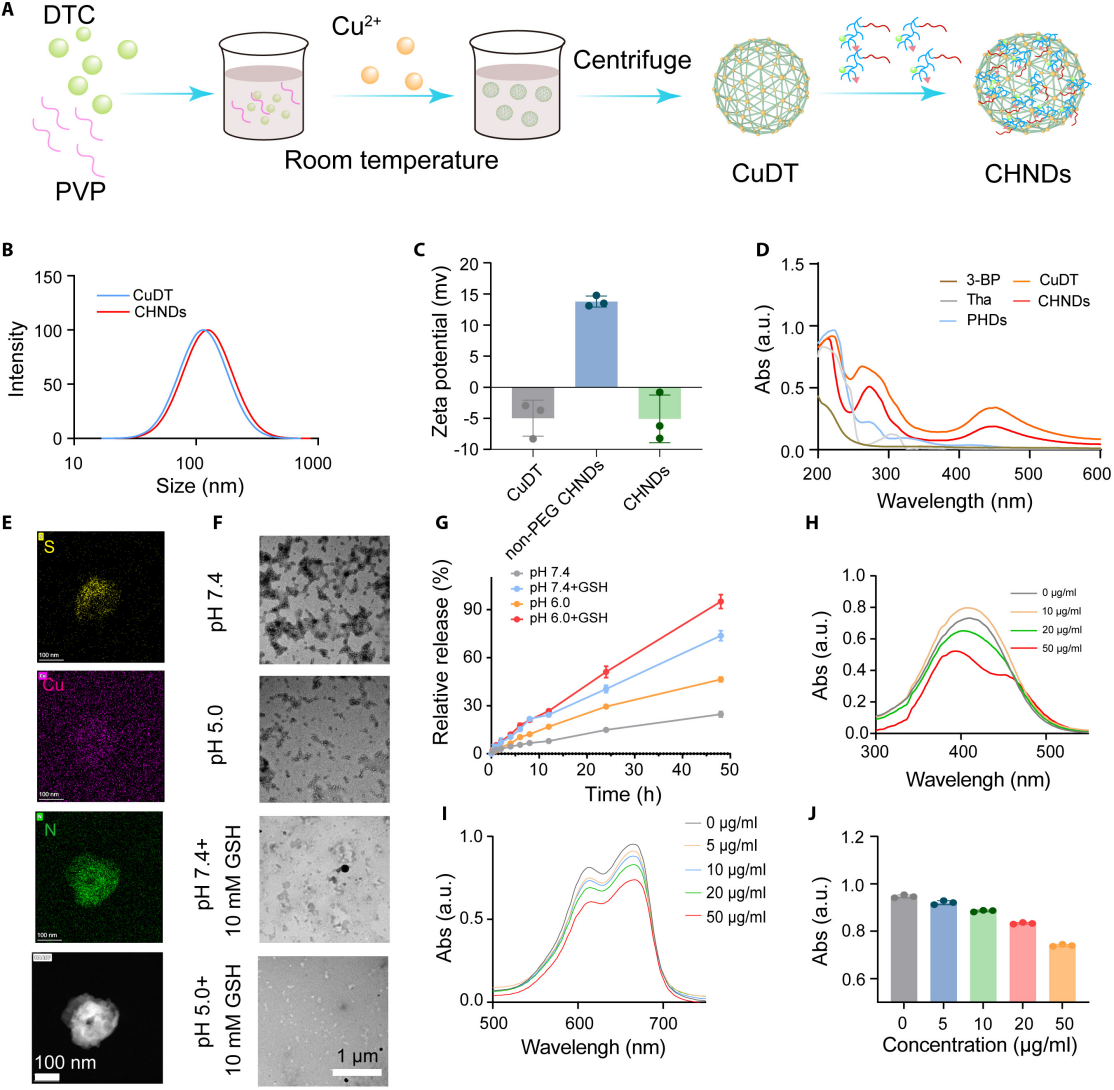
Preparation and characterization of CHNDs. (A) Schematic of CHNDs preparation. (B) Hydrodynamic diameter and particle distribution of CuDT and CHNDs detected by DLS. (C) Zeta potential of CuDT, non-PEG CHNDs, and CHNDs. (D) UV–Vis spectra of 3-BP, CuDT, THA, PEG-PHDs, and CHNDs. (E) Transmission electron microscopy (TEM) images of CHNDs and the corresponding elemental distribution maps of S, Cu, and N. Scale bar, 100 nm. (F) TEM images of CHNDs in pH 7.4/pH 5.0 PBS with or without 10 mM GSH. Scale bar, 1 μm. (G) Cumulative release profiles of PEG-PHDs from CHNDs dispersed in pH 7.4/pH 6.0 PBS with or without 10 mM GSH at 37 °C. (H) Detection of GSH consumption CHNDs by UV–Vis spectra scanning using DTNB as a probe. (I) UV–Vis spectra of MB solutions ranged from 500 to 750 nm after treatment with CHNDs and H_2_O_2_. (J) Statistical quantification of absorbance of MB solutions at 675 nm after co-incubation of MB with CHNDs and H_2_O_2_.

Metal–organic coordination polymers are perfect drug carrier owing to its porous structure, which have already been used to deliver drugs in several studies [46]. Herein, we prepared the copper-DTC complex nanoparticles (CuDT NPs) by metal–organic coordinate function between diethyldithiocarbamate (DTC) and Cu^2+^ at a molar ratio of 2:1, via a facile one-pot self-assembly profile [47]. Then, we designed and prepared multifunctional nanoscale Cu-MOF-based selective HK-2 nano-degraders (CHNDs) via a simple layer-by-layer method under ambient conditions due to electrostatic and hydrophobic interaction (Fig. 3A). The crystal structure of CuDT and CHNDs was investigated by the powder x-ray diffraction (XRD) pattern. As depicted in Fig. S5, the XRD pattern of both CuDT and CHNDs exhibited several distinct diffraction peaks, indicative of the crystalline nature of these nanomaterials. In addition, we conducted a series of experiments to characterize CuDT/CHNDs, including x-ray photoelectron spectroscopy (XPS) and N_2_ adsorption–desorption isotherms (BET). A Cu 2p binding energy peak in the XPS spectrum of non-PEG CHNDs demonstrated that Cu was doped in the non-PEG CHNDs. The binding energies attributed to Cu 2p_3/2_ and Cu 2p_1/2_ were observed in the high-resolution Cu 2p spectrum, illustrating the existence of Cu^2+^ in the non-PEG CHNDs (Fig. S6). The N_2_ adsorption–desorption and pore size distribution profiles of CHNDs were detected, as shown in Fig. S7. CHNDs exhibited characteristic type IV hysteresis loops representing mesoporous structures, and the pore size was 4.37 nm. The hydrodynamic diameter of CuDT and CHNDs was detected by the dynamic light scattering (DLS). As indicated by quantitative DLS characterization, after CuDT was coordinated with PEG-PHDs, the average diameter of CHNDs exhibited a slight increase from 142.2 ± 8.26 nm (bare CuDT) to 158.4 ± 6.9 nm (Table S1 and Fig. 3B). Both CuDT and CHNDs NPs had favorable stability in either phosphate-buffered saline (PBS) or RPMI 1640 medium with 10% fetal bovine serum (FBS), demonstrating no remarkable size change after incubation for 48 h (Fig. S8). The surface charge of non-PEG CHNDs NPs was changed from −4.87 mV to 13.78 mV after coating with PHDs, demonstrating the successful decoration of PHDs on the CuDT NPs. The zeta potential further changed to negative charge after conjugation with the PEGylation derivative (Fig. 3C). PEGylation decoration conferred CHNDs with slight negative zeta potential and satisfactory physiological stability. The successful preparation of CHNDs was further demonstrated by parallel characteristic peaks of PHDs at about 218 nm and CuDT at 440 and 270 nm in the ultraviolet–visible (UV–Vis) spectrum of CHNDs (Fig. 3D).

Transmission electron microscopy (TEM) observations revealed that CHNDs possessed a well-defined, homogeneous sheet-shaped structure, with an average size of about 150 nm. As analyzed by energy-dispersive spectrometry (EDS) and elemental localization analyses (Fig. 3E and Fig. S9), the as-prepared CHNDs exhibited distinct element signals of Cu, S, and N. The decomposition behavior of CHNDs was visually detected by TEM. As illustrated in Fig. 3F, under the microenvironment of pH 5.0 containing 10 mM glutathione (GSH), the TEM images revealed distinct structural disassembly of CHNDs, demonstrating that the CHNDs exhibited pH/redox-responsive disintegration capabilities under acidic and high-reductive GSH condition. The structure of CHNDs began to decompose at pH 5.0, with edges becoming blurred. CHNDs almost lost its nanostructure at pH 5.0 + GSH, which should be attributed to the disassembly of copper ion–DTC coordination at acidic pH and redox condition. Thereafter, the release profiles from the CHNDs were subsequently investigated under both simulated physiological conditions (pH 7.4) and a reductive milieu mimicking the tumor microenvironment (pH 6.0, 10 mM GSH). As depicted in Fig. 3G, 24.7% PEG-PHDs was liberated in PBS at pH 7.4. In comparison, under the mildly acidic and reductive conditions (pH 6.0, 10 mM GSH), the extent of PEG-PHDs release reached 95.1% after 48 h, representing a substantial 2.6-fold increase compared to that observed at pH 7.4, which could be ascribed to GSH-triggered weakened coordination interactions and the protonation of PEG-PHDs. This enhanced release profile under tumor-relevant conditions underscores the potential of the CHNDs for targeted drug delivery.

Moreover, since GSH endowed cancer cells with the anti-cuproptosis capacity and acted as a ROS scavenger, the GSH-scavenging capacity of CHNDs was evaluated using 5,5′-dithiobis-(2-nitrobenzoic acid) (DTNB) as a chromogenic probe, where DTNB could react with GSH to yield the maximum absorbance at 412 nm of the 2-nitro-5-thiobenzoate (TNB) anion. As depicted in Fig. 3H, the addition of CHNDs to a GSH solution resulted in a marked decrease in the absorbance of DTNB at 412 nm with the concentration of CHNDs. This attenuation in absorbance signified the consumption of GSH through its interaction with the CHNDs, thus corroborating their GSH-scavenging properties. This observation suggested a potential mechanism by which CHNDs could upset intracellular redox homeostasis through the depletion of GSH. Prior studies had demonstrated that Cu^+^ reduced by GSH could exhibit dramatically higher catalytic activities than Cu^2+^, consequently driving Fenton-like reactions and enhancing the generation of hydroxyl radicals (•OH) [4]. To corroborate the hypothesized role of Cu^+^ in •OH production by CHNDs in the presence of H_2_O_2_, methylene blue (MB) assay was employed as a probe to quantify •OH generation. Upon incubation of MB with CHNDs and H_2_O_2_, a substantial decrease in the distinctive absorbance peak of MB at 665 nm was observed, diminishing from 0.95 to 0.74 with the increased concentration of CHNDs (Fig. 3I and J). This spectral shift strongly suggested the generation of •OH by the CHNDs with H_2_O_2_ at acid and redox condition, consistent with the proposed Cu^+^-mediated Fenton-like mechanism. The preceding results demonstrated that the CHNDs were expected to reduce unintended cytotoxicity toward normal cells while being selectively activated in tumor cells through their tumor-specific cascade catalytic activity. Overall, we had successfully established an innovative engineered nano-protein degrader platform, designated CHNDs, for potential targeted protein degradation and cancer therapy within the tumor microenvironment.

### CHNDs effectively provoked HK-2 degradation in vitro

**Fig. 4.**
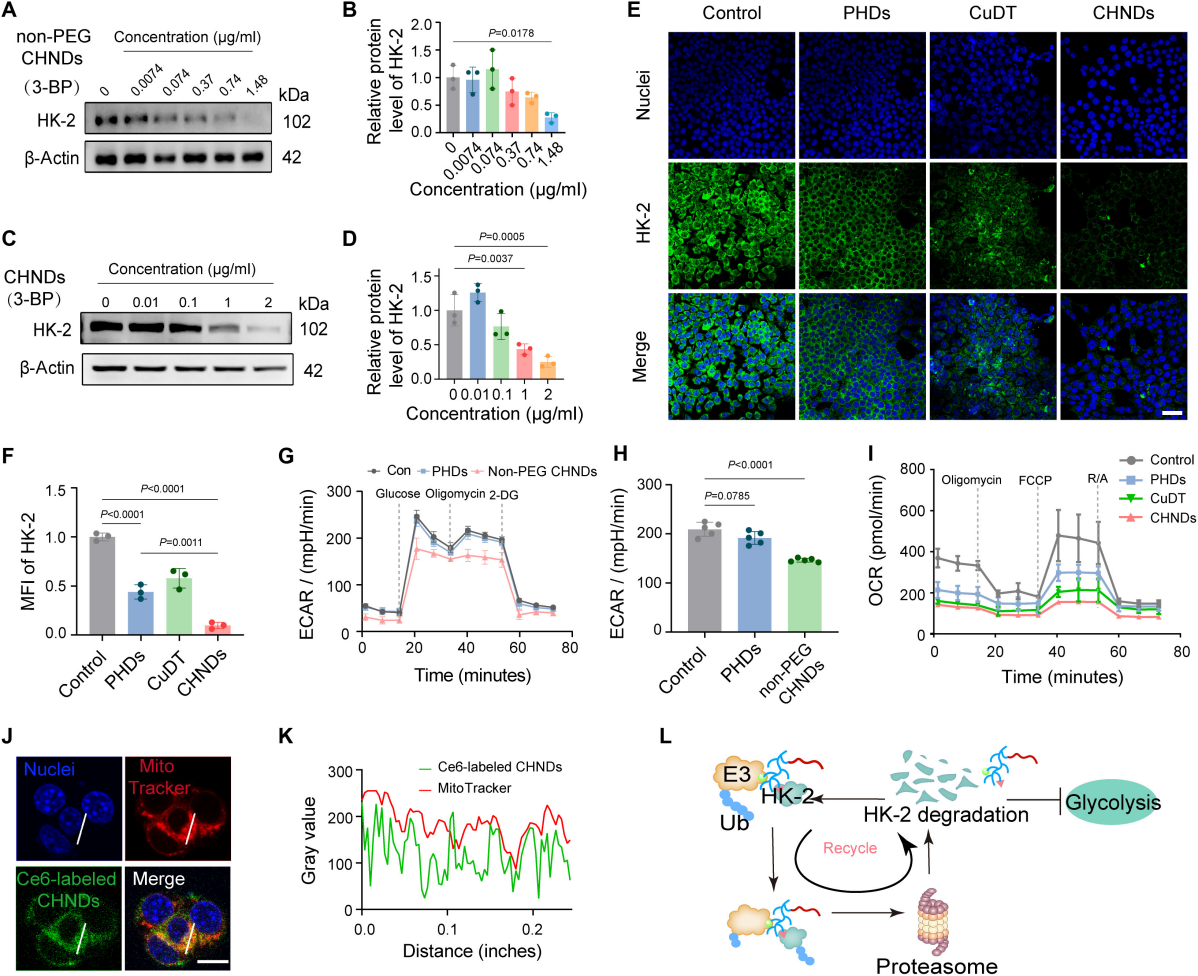
CHNDs degraded HK-2 in tumor cells and inhibited glycolysis. (A) Concentration-dependent HK-2 degradation in 4T1 cells following 24 h of non-PEG CHNDs treatment at varying concentrations. (B) Quantitative statistical result of gray value for (A) graph was quantified by ImageJ. β-Actin was employed as the control reference. (C) WB analysis of HK-2 proteins in 4T1 cells following 24 h of CHNDs treatment at varying concentrations. (D) Grayscale intensity values in (C) were quantified using ImageJ. β-Actin was used as the loading control. (E) After 24 h of exposure to PHDs, CuDT, or CHNDs, 4T1 cells were subjected to HK-2-specific immunofluorescent staining. Scale bar, 50 μm. (F) Fluorescence intensity was subsequently quantified using ImageJ. (G) The extracellular acidification rate (ECAR) of tumor cells was measured after 12 h of exposure to PBS, PHDs, or non-PEG CHNDs. (H) Quantification of glycolysis in 4T1 cells treated with PBS, PHDs, or non-PEG CHNDs. (I) Oxygen consumption rate (OCR) profile of 4T1 upon treatment with different groups. (J) Intracellular localization of CHNDs in 4T1 cells following incubation with Ce6-labeled CHNDs and MitoTracker. Scale bar, 10 μm. Blue, 4′,6-diamidino-2-phenylindole (DAPI); red, MitoTracker; green, Ce6-labeled CHNDs. (K) Quantification of colocalization between CHNDs and MitoTracker based on grayscale intensity analysis. (L) Schematic illustration of PEG-PHDs-mediated HK-2 degradation. All significance analyses in this figure were performed using one-way ANOVA.

Following the preparation of the non-PEG CHNDs and CHNDs, the HK-2 degradation capacity of non-PEG CHNDs and CHNDs in tumor cells was assessed by WB analysis. As shown in Fig. S10, HK-2 degradation exhibited a clear time-dependent pattern following treatment with non-PEG CHNDs, with overexpressed HK-2 levels progressively declining as incubation time increased. Quantitatively, treatment with non-PEG CHNDs reduced HK-2 expression in 4T1 cells to 27.6% of control levels. As illustrated in Fig. 4A and B, treatment with non-PEG CHNDs nanodrugs resulted in a substantial concentration-responsive degradation in HK-2 protein levels compared with control levels (*P* = 0.0178). In accordance with the foregoing results, non-PEG CHNDs exhibited a similar time- and concentration-dependent down-regulation of HK-2 in CT26 cells, with HK-2 levels decreasing to 27.5% compared to control groups (*P* = 0.0075) (Figs. S11 and S12). These results collectively demonstrated the potent HK-2 degradation activity of non-PEG CHNDs in both 4T1 and CT26 cancer cell lines.

To endow the long blood circulation characteristics and avoid in vivo limitations of the cationic polymer PHDs including nephrotoxicity, immunogenicity, and other potential adverse effects [48], we employed a strategy of PEGylation to mitigate the dilemma. Specifically, PHDs was conjugated with mPEG_2k_ derivative to yield the biocompatible copolymer PEG-PHDs (Fig. 2B). Then, PEG-PHDs was incorporated into the design of multifunctional copper-based nano-PROTACs (CHNDs). To investigate the influence of PEGylation derivative modification on the HK-2 degradation efficacy of CHNDs, we compared the ability of non-PEG CHNDs and CHNDs on HK-2 degradation in 4T1 cells using WB and immunofluorescence staining assay. WB analysis demonstrated that CHNDs still displayed excellent degradation effect on HK-2 protein in a concentration-responsive pattern (Fig. 4C and D). This was also verified by immunofluorescent staining of HK-2, revealing that CHNDs kept the capacity to degrade HK-2 in 4T1 cells even in the incorporation of PEG derivative on the PHDs, as depicted in Fig. 4E. Semiquantitative analysis of the immunofluorescence staining images revealed a striking reduction in intracellular HK-2 levels upon treatment with CHNDs, reaching 9.7% of control levels (*P* < 0.0001) (Fig. 4F). The results shown in Fig. S13 further confirmed the HK-2 degradation effect induced by PEG-PHDs.

Encouraged by the efficacy of the CHNDs in down-regulating cellular HK-2, we next investigated the consequential impact on glycolytic activity, given the central role of HK-2 in tumor cell glycolysis. The extracellular acidification rate (ECAR), a well-established indicator of glycolytic flux, was measured using a Seahorse XF Analyzer. As shown in Fig. 4G, treatment of 4T1 cells with non-PEG CHNDs resulted in a substantial suppression of glycolysis compared to the PBS group and cells treated with PHDs alone. Quantitative analysis revealed a significant attenuation in glycolytic capacity, decreasing by 30.0% and 21.6% in non-PEG CHNDs-treated cells compared to control and PHDs-treated groups, respectively (Fig. 4H and Fig. S14). Moreover, maximal glycolysis was diminished by 23.7% in the non-PEG CHNDs group compared to untreated cells. Furthermore, we conducted seahorse mitochondrial stress tests to evaluate mitochondrial respiration. Compared to the control group, PHDs and CuDT significantly reduced basal respiration, adenosine triphosphate (ATP) production, maximal respiration capacity (Fig. 4I). Taken together, these data unequivocally demonstrated the potent inhibitory effect of CHNDs on glycolysis and mitochondrial respiration in 4T1 tumor cells, likely attributable to the observed down-regulation of HK-2. Given the established localization of HK-2 protein to the outer mitochondrial membrane [17], we investigated the mitochondrial targeting capability of CHNDs. 4T1 cells were incubated with CHNDs incorporating Ce6-labeled PEG-PHDs to facilitate visualization. Confocal microscopy analysis, as presented in Fig. 4J and K, revealed a high degree of spatial colocalization between the fluorescence intensity profiles of Ce6-labeled CHNDs and MitoTracker within the indicated regions. The extent of colocalization between mitochondria and the CHNDs was quantified using Pearson’s correlation coefficient and Mander’s colocalization coefficients, as presented in Fig. S15. This colocalization strongly suggested the efficient targeting of CHNDs to the mitochondria, evidencing their potential to directly interact with and degrade HK-2 at its primary site of action.

The proposed mechanism of action for PEG-PHDs, subsequent to its release from the CHND delivery system, was illustrated in Fig. 4L. In essence, PEG-PHDs functioned as a targeted protein degradation agent by acting as a bridge between HK-2 and the cellular UPS. Specifically, PEG-PHDs bound directly to HK-2, simultaneously recruiting an E3 ubiquitin ligase. This ternary complex formation brought HK-2 into close proximity with the E3 ligase, facilitating the ubiquitination of HK-2. Subsequently, the ubiquitin-tagged HK-2 was recognized and degraded by the 26S proteasome, releasing PEG-PHDs to initiate further cycles of targeted HK-2 degradation. This proposed mechanism highlighted the potential of PEG-PHDs as a catalytic degrader of HK-2.

### Cuproptosis induced by CHNDs

**Fig. 5.**
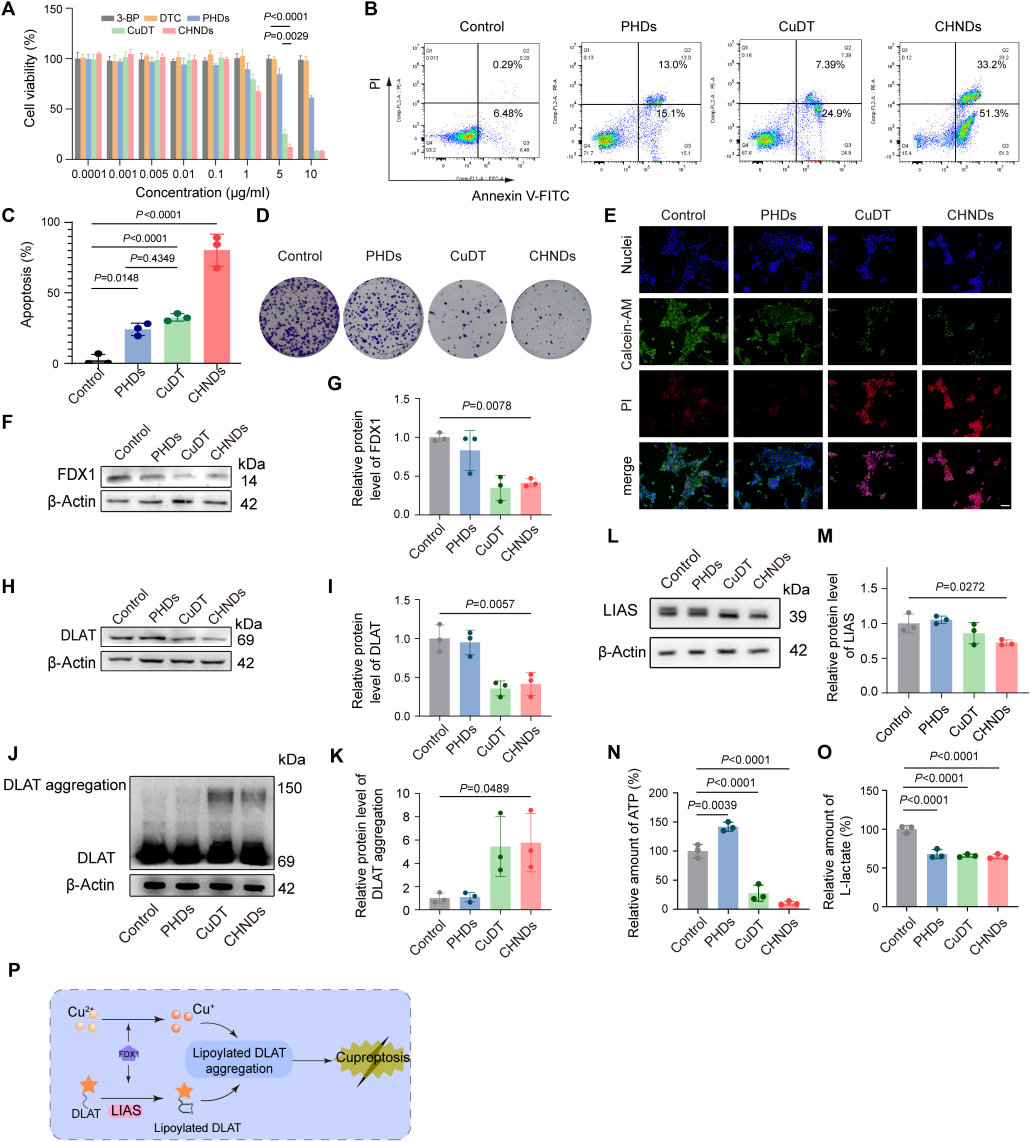
CHNDs provoked cuproptosis in tumor cells. (A) After incubating 4T1 cells with 3-BP, DTC, PHDs, CuDT, or CHNDs for 48 h, cell viability at various treatment concentrations was assessed using the MTT assay. (B) The apoptosis ratio of 4T1 cells upon treatment with PHDs, CuDT, and CHNDs was detected by flow cytometric apoptosis analysis. (C) The flow cytometric apoptosis analysis was statistically quantified. (D) The colony formation assay was utilized to evaluate the inhibitory activity of PHDs, CuDT, and CHNDs on the proliferation of 4T1 cells. (E) After incubating 4T1 cells with PHDs, CuDT, and CHNDs for 24 h, cell viability was detected by live/dead cell staining assay. (F) Expression of FDX1 by WB. (G) ImageJ quantitative statistical gray value of FDX1; β-actin as quantitative internal reference. (H) The protein level of DLAT was detected by WB. (I) The protein levels of DLAT were quantified by ImageJ, and β-actin was used as a loading control. (J) WB analysis of DLAT aggregation in 4T1 cells treated with different drugs for 24 h (3-BP: 1 μg/ml). (K) Quantification of DLAT aggregation by ImageJ, and β-actin was used for control. (L) The protein level of LIAS was detected by WB. (M) LIAS expression levels were quantitatively analyzed using ImageJ, with β-actin serving as the loading control for sample normalization. (N) Detection of intracellular ATP content after treatment with different treatments. (O) Determination of l-lactic acid content in cells after treatment with different formulations. (P) Schematic diagram of the mechanism of CHNDs promoting cuproptosis in tumor cells. All significance analyses in this figure were performed using one-way ANOVA.

HK-2, a critical regulator of tumor metabolism, exerted control over glycolysis, while copper played an essential role in maintaining mitochondrial function [5]. Herein, the engineered CHNDs platform achieved synergistic dual inhibition of tumor metabolism by integrating PEG-PHDs-mediated HK-2 degradation with cuproptosis induction through the spatiotemporally controlled release of copper ions from the CuDT core. To evaluate the therapeutic efficacy of this strategy, the in vitro cytotoxicity of PHDs, CHNDs, 3-BP, and DTC against 4T1 cells was assessed by 3-(4,5-dimethylthiazol-2-yl)-2,5-diphenyltetrazolium bromide (MTT) assays. As shown in Fig. 5A, both PHDs and CHNDs exhibited significantly enhanced cytotoxicity toward 4T1 cells, whereas 3-BP and DTC showed only moderate growth inhibition. Notably, treatment with CHNDs reduced cell viability to 12.2% at an equivalent 3-BP concentration of 5 μg/ml, suggesting a synergistic contribution of glycolysis inhibition and cuproptosis. Consistently, CHNDs exhibited a lower IC_50_ (median inhibitory concentration) value (1.34 μg/ml) than CuDT alone (2.67 μg/ml), further indicating enhanced antitumor potency. The cytotoxicity of CuDT and CHNDs at high concentration of copper ions showed no statistically significant difference, because the pronounced intrinsic cytotoxicity of CuDT may mask the additional contribution from PHDs. Apoptosis analysis by Annexin V–fluorescein isothiocyanate (FITC)/propidium iodide (PI) staining confirmed this trend (Fig. 5B and C), with CHNDs inducing 80.4% total apoptosis, corresponding to a 2.47-fold increase compared to the CuDT group (*P* < 0.0001). Colony formation assays further demonstrated the strong antiproliferative effect of CHNDs (Fig. 5D). Quantitative analysis supported the potent inhibitory effect of CHNDs on 4T1 cell proliferation (Fig. S16). Collectively, these results demonstrated that the combination of cuproptosis induction and glycolysis inhibition synergistically enhanced tumor cell eradication.

To further examine the effect of CHNDs on cellular redox homeostasis, intracellular ROS levels in 4T1 cells were evaluated using 2′,7′-dichlorofluorescein diacetate (DCFH-DA). As shown in Fig. S17, treatment with PHDs, CuDT, or CHNDs led to a marked increase in intracellular ROS levels, suggesting that the observed cytotoxicity is partially associated with redox imbalance. Given the pivotal role of HK-2 in maintaining mitochondrial integrity and membrane potential, mitochondrial function was further assessed using the JC-1 assay. As shown in Fig. S18A, untreated 4T1 cells exhibited predominant red fluorescence, indicative of intact mitochondrial membrane potential and normal rod-like mitochondrial morphology. In contrast, cells treated with PHDs, CuDT, or CHNDs displayed a pronounced increase in green fluorescence, reflecting mitochondrial membrane depolarization. Notably, CHNDs treatment resulted in almost complete disruption of the rod-like mitochondrial structure and substantially higher green fluorescence intensity compared with the other treatment groups, indicating more severe mitochondrial damage. Quantitative analysis of the JC-1 monomer-to-aggregate ratio (Fig. S18B) revealed 1.7-fold, 1.5-fold, and 2.4-fold increases for PHDs, CuDT, and CHNDs treatments, respectively, relative to the control group, with CHNDs showing the most pronounced mitochondrial depolarization (*P* = 0.0407). Consistently, live/dead cell staining using calcein-AM and PI further confirmed the potent cytotoxicity of CHNDs (Fig. 5E), as evidenced by intense PI-associated red fluorescence following treatment.

Copper overabundance within tumor cells had been shown to trigger cuproptosis [49], a paradigm of programmed cell death. To evaluate whether CHNDs induce cuproptosis, dihydrolipoamide S-acetyltransferase (DLAT) oligomerization was examined by immunofluorescence staining following different treatments. As shown in Fig. S19, CHNDs treatment resulted in a pronounced increase in DLAT aggregation compared with the other groups, indicating effective induction of cuproptosis. To further elucidate the underlying mechanism, the expression levels of key cuproptosis-related proteins were analyzed. WB analysis revealed that CuDT and CHNDs treatments significantly reduced the protein levels of FDX1 (*P* = 0.0087) and DLAT (*P* = 0.0057) compared with the control group (Fig. 5F to I), whereas PHD treatment showed no detectable effect. Quantitatively, FDX1 expression was reduced to 0.35- and 0.41-fold of control levels following CuDT and CHNDs treatment, respectively, consistent with copper-induced destabilization of cuproptosis-associated proteins. In addition, WB analysis further confirmed DLAT oligomerization, with the appearance of high-molecular-weight DLAT bands (~150 kDa), accompanied by attenuation of lipoic acid synthetase (LIAS) expression following CuDT and CHNDs treatment (Fig. 5J to M). These results collectively demonstrated that CHNDs triggered cuproptosis through dysregulation of the FDX1–DLAT–LIAS axis, similar to CuDT.

Given the simultaneous degradation of HK-2 and induction of cuproptosis, intracellular energy metabolism was further evaluated. As shown in Fig. 5N, CHNDs treatment significantly reduced intracellular ATP levels compared with the control group (*P* < 0.0001). Meanwhile, lactate production was markedly decreased following CHNDs treatment (*P* < 0.0001; Fig. 5O), indicating effective suppression of glycolytic activity. A schematic illustration of the proposed cuproptosis mechanism induced by CHNDs is presented in Fig. 5P. Upon cellular internalization, the copper ions were reduced by FDX1, facilitating DLAT lipoylation and subsequent copper-mediated DLAT oligomerization, ultimately leading to mitochondrial dysfunction and cuproptotic cell death.

**Fig. 6.**
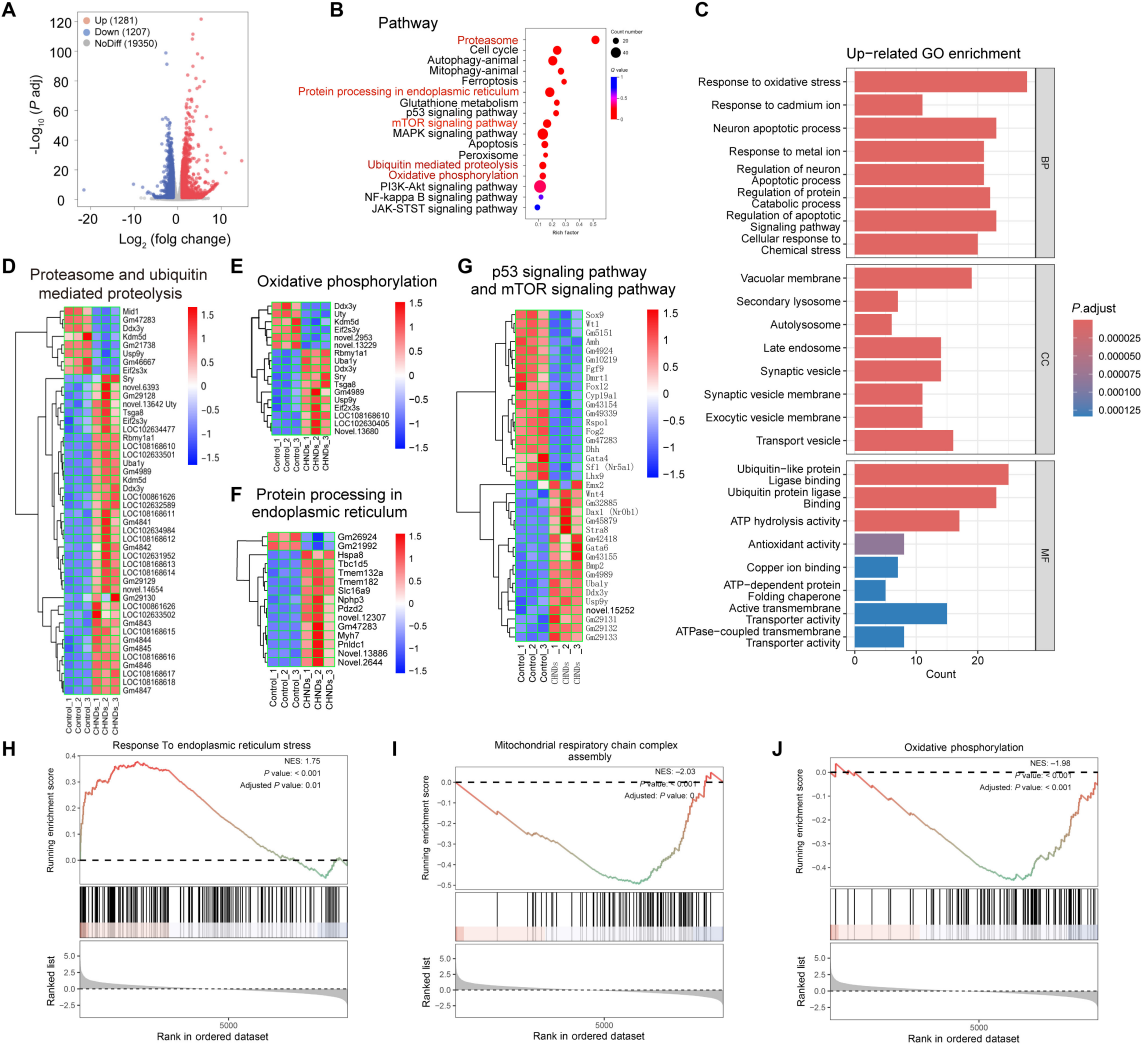
RNA-sequencing analysis of 4T1 cells treated with PBS and CHNDs. (A) Volcano plots displayed the differentially expressed genes in cells treated with CHNDs compared to control groups. (B) KEGG analysis of differentially expressed genes between cells treated with PBS and CHNDs. (C) GO enrichment analysis of up-regulated transcripts. Heatmap of proteasome and ubiquitin-mediated proteolysis (D), oxidative phosphorylation cascades (E), protein processing in endoplasmic reticulum (F), and the p53–mTOR signaling axis (G) gene expressions in cells treated with PBS and CHNDs. (H) Gene set enrichment analysis (GSEA) revealed significant enrichment of endoplasmic reticulum stress response-related gene sets in CHNDs-treated cells. (I and J) GSEA further demonstrated that gene sets associated with mitochondrial respiratory chain complex assembly and oxidative phosphorylation were significantly down-regulated following CHNDs treatment.

Our investigation evaluated the intracellular abundance of DLAT and FDX1 proteins following CHNDs administration, providing preliminary validation of CHNDs-induced copper-mediated cytotoxicity. Subsequent transcriptomic change analysis via the genome-wide RNA sequencing revealed distinctive alterations in cellular gene expression profiles upon CHNDs treatment. Compared with PBS-treated cells, CHNDs-treated 4T1 cells exhibited 1,281 significantly up-regulated genes and 1,207 significantly down-regulated genes (Fig. 6A). Gene Ontology (GO) and Kyoto Encyclopedia of Genes and Genomes (KEGG) enrichment analyses were performed to examine functional pathway alterations in CHNDs-treated 4T1 cells. KEGG analysis revealed significant up-regulation of the proteasome, protein processing in the endoplasmic reticulum, ubiquitin-mediated proteolysis, mammalian target of rapamycin (mTOR) signaling pathway, and oxidative phosphorylation following CHNDs exposure (Fig. 6B). Besides, molecular indicators associated with antioxidant defense mechanisms and copper ion binding demonstrated marked enrichment, potentially reflecting cellular adaptive responses to CHNDs-induced copper ion-mediated cytotoxicity. The analysis further revealed enhanced adenosine triphosphatase (ATPase)-coupled transmembrane transporter activity, suggesting CHNDs-mediated perturbation of cellular bioenergetics and membrane transport processes.

GO enrichment analysis of up-regulated transcripts (Fig. 6C) revealed significant enrichment across multiple biological processes following CHNDs administration. Notably, oxidative stress response pathways exhibited pronounced enrichment, indicating robust activation of cellular oxidative damage response mechanisms. Substantial enrichment was also observed in calcium ion response pathways, suggesting significant modulation of metal ion stress response mechanisms under the experimental conditions. Moreover, enrichment of cancer cell apoptotic processes and their regulatory pathways implies CHNDs-mediated modulation of programmed cell death mechanisms.

Differential expression analysis (Fig. 6D to G) revealed significant transcriptional modulation of genes involved in the pathway of proteasome and ubiquitin-mediated proteolysis, oxidative phosphorylation cascades, protein processing in endoplasmic reticulum, and the p53–mTOR signaling axis following CHNDs treatment. Specifically, Fe–S cluster genes and TCA cycle-associated genes were down-regulated. Furthermore, the endoplasmic reticulum stress response pathway was significantly enriched in CHNDs-treated 4T1 cells, as revealed by gene set enrichment analysis (GSEA) (Fig. 6H), while the mitochondrial respiratory chain complex assembly and oxidative phosphorylation pathway were found to be significantly suppressed in 4T1 cells treated with CHNDs by GSEA (Fig. 6I and J). Collectively, the evidence demonstrated that CHNDs administration induced substantial disruptions in cellular redox homeostasis, metabolic processes, and ubiquitin–proteasome pathway function.

### In vivo potent anticancer efficacy of CHNDs via cuproptosis induction and glycolysis inhibition

**Fig. 7.**
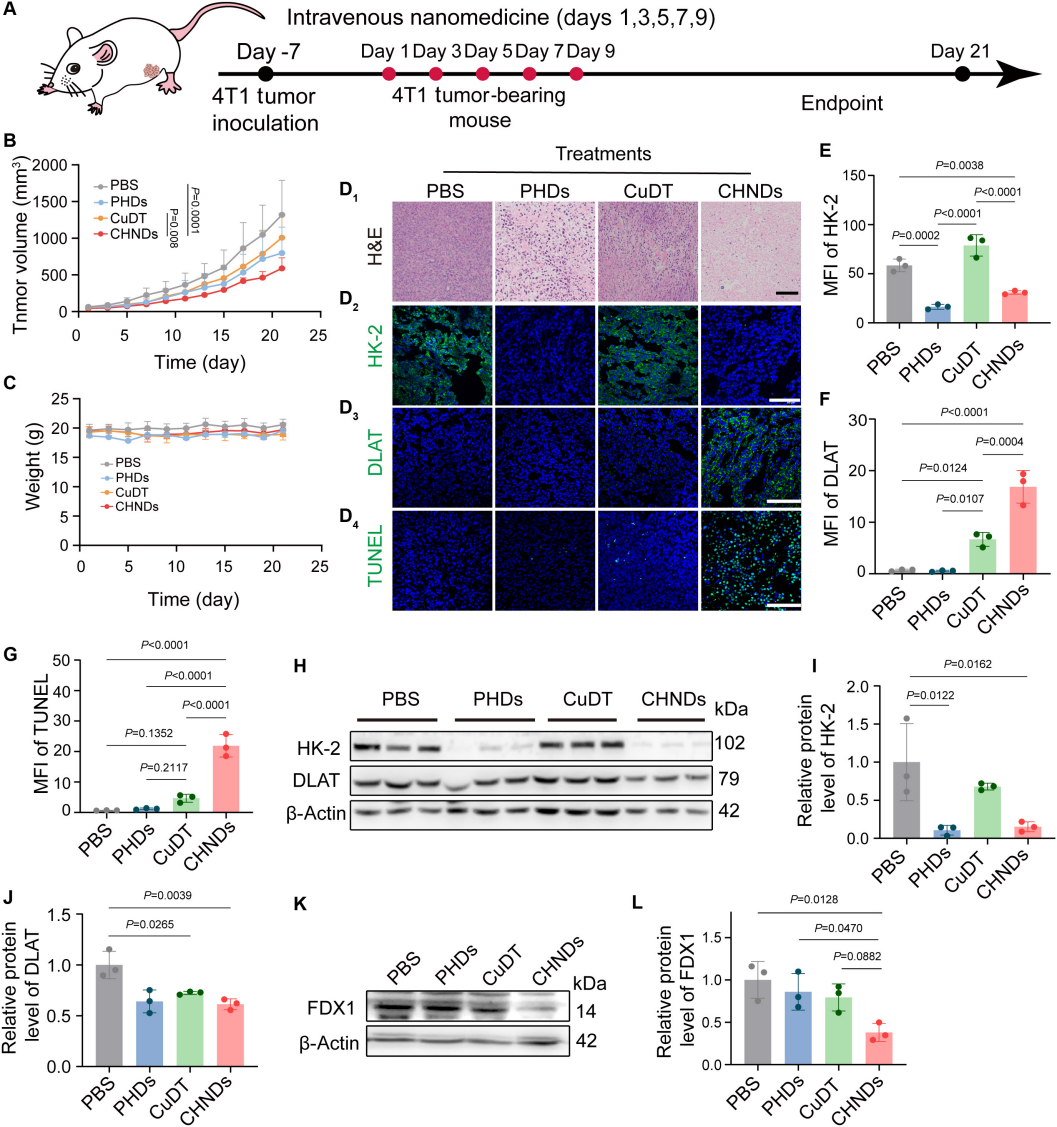
Antitumor efficacy of CHNDs in 4T1 tumor-bearing mice in vivo. (A) Schematic overview of the treatment schedule for antitumor efficacy assessment. When 4T1 tumors reached a volume of 50 to 100 mm^3^, mice received 5 intravenous administrations of the designated treatments. Tumor volumes and body weights were measured at regular intervals to evaluate therapeutic efficacy (*n* = 5). Tumor-bearing mice were treated with PBS, PHDs (2 mg 3-BP/kg), CuDT (4.5 mg CuDT/kg), or CHNDs (2 mg 3-BP/kg and 4.5 mg CuDT/kg). (B) Tumor growth curve after different treatments. (C) Body weight changes of 4T1 tumor-bearing mice treated with PBS, PHDs, CuDT, and CHNDs. (D_1_ to D_4_) H&E staining and representative confocal images (green: HK-2, DLAT, and TUNEL; blue: nuclei) of 4T1 tumor tissues. Scale bar, 100 μm. Mean fluorescence intensity (MFI) quantification of HK-2 (E), DLAT (F), and TUNEL (G). (H) WB analysis of HK-2 and of cuproptosis-associated proteins (such as DLAT) in tumor tissues. Quantitative analysis of HK-2 (I) and DLAT (J) using ImageJ. β-Actin was used as control. (K) WB analysis of FDX1 in 4T1 tumor tissues. (L) Semiquantification of FDX1 by ImageJ. The data in (B) were analyzed using two-way ANOVA, while the remaining quantitative analyses were performed using one-way ANOVA.

Motivated by the promising in vitro cytotoxic activity of CHNDs, we further evaluated the antitumor efficacy of this synergistic therapeutic strategy in vivo. An orthotopic breast cancer model was established using 4T1 cells. The treatment regimen was illustrated in Fig. 7A. PBS, PHDs, CuDT, or CHNDs were administered to tumor-bearing mice via tail vein injection for 5 consecutive doses at 2 mg 3-BP/kg and 4.5 mg CuDT/kg. Tumor volumes and body weights were measured and recorded every other day. As depicted in Fig. 7B, tumor growth upon treatment with CHNDs was significantly inhibited compared to the PHD, CuDT, or PBS group. Quantitative analysis revealed a remarkable tumor growth inhibition rate of 55.3% in the CHNDs group compared to the control group (*P* = 0.0001), highlighting its potent antitumor efficacy. The superior antitumor efficacy of CHNDs was also intuitively observed from the representative images of the tumor-bearing mice (Fig. S20). These results collectively demonstrated that the combined therapeutic modalities of CHNDs provided a synergistic benefit in suppressing tumor growth due to the aggravated cuproptosis and glucose metabolism reprogramming.

Following investigating potent antitumor efficacy, the biosafety of CHNDs was validated in BALB/c mice via intravenous injection with 5 doses, showing stable body weights and hematological parameters (Fig. 7C and Fig. S21). No obvious major organ damaged was observed except for the PHDs treatment (Fig. S22). The results corroborated our prior concerns regarding the potential hepatotoxicity of PHDs, as liver inflammation and elevated neutrophil and monocyte counts in the deceased animals. This observation underscored the potential risks associated with systemic administration of PHDs and highlighted the necessity for PEGylation decoration to mitigate the toxicity of cationic polymer PEI. In addition, the negligible hemolysis rate implied the suitability of CHNDs for injectable drug administration (Fig. S23).

Histological analysis of tumor tissues further corroborated the in vivo antitumor efficacy of CHNDs. Hematoxylin and eosin (H&E) and terminal deoxynucleotidyl transferase-mediated deoxyuridine triphosphate nick end labeling (TUNEL) staining demonstrated more pronounced apoptotic and necrotic changes in tumor tissues from the CHNDs group compared with the other treatment groups (Fig. 7D). Immunofluorescence staining for HK-2 further revealed significantly reduced HK-2 expression in both the PHDs and CHNDs groups relative to the PBS control (Fig. 7D). Quantitative analysis revealed a substantial 47.2% decrease in HK-2 levels in the CHNDs group compared to the PBS group (*P* = 0.0038) (Fig. 7E), confirming the effective degradation of HK-2 protein in vivo. Furthermore, immunofluorescence staining of DLAT (Fig. 7D) showed slight DLAT aggregation in the CuDT group (*P* = 0.0124) and pronounced aggregation in the CHNDs group (*P* < 0.004) compared to the PBS group, indicative of cuproptosis induction induced by CHNDs. Quantitative analysis of DLAT aggregation (*P* < 0.0001) and TUNEL-positive areas (*P* < 0.0001) in tumor tissue compared to the PBS group further collaborated the significant induction of cuproptosis and apoptosis in the CHNDs group (Fig. 7F and G). Taken together, these histological and immunofluorescence results demonstrated that CHNDs treatment effectively induced tumor tissue apoptosis and cuproptosis, exerting the observed tumor growth inhibition. To further quantify the effects of the treatments on pivotal proteins involved in tumor glycolysis and copper toxicity-related proteins, we performed WB analysis on extracting proteins from tumor tissue lysates. As shown in Fig. 7H to J, HK-2 was significantly down-regulated upon treatment with PHDs (*P* = 0.0122) and CHNDs groups (*P* = 0.0162) compared to the PBS group, with eradication by 89.3% and 84.8%, respectively. DLAT protein was also decreased in the tumor tissues, decreasing by 27.5% and 38.6% in the CuDT and CHNDs treatment groups, respectively. Additionally, consistent with our in vitro observations, FDX1 protein dysregulation was observed in the CHNDs treatment group (Fig. 7K and L), further providing evidence for the involvement of cuproptosis after treatment with CHNDs.

**Fig. 8.**
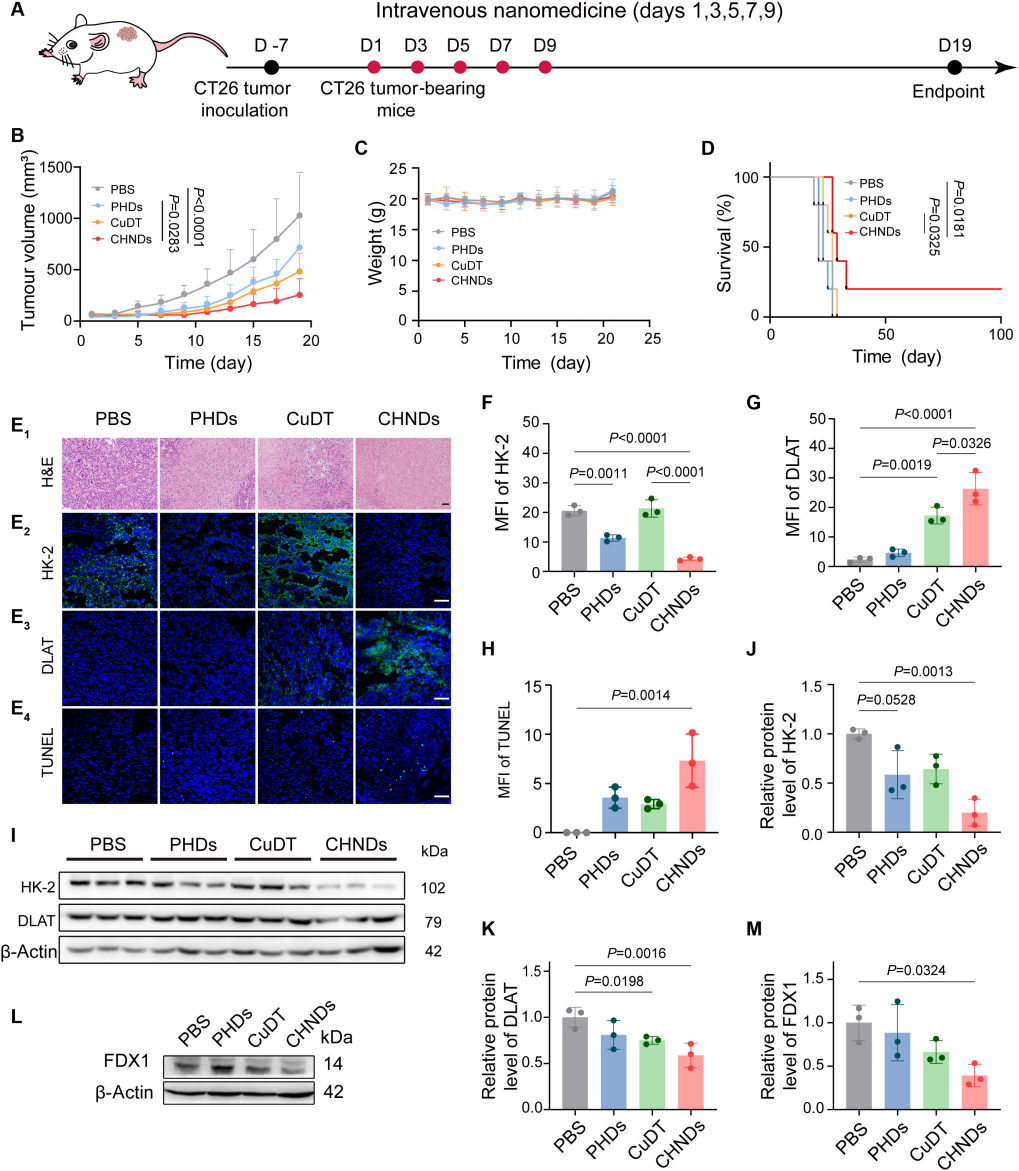
Effect of CHNDs on inhibiting tumor growth in vivo. (A) Scheme illustrating the experiment dosage regimen. When CT26 tumors reached 50 to 100 mm^3^, mice received 5 drug treatments, and tumor volumes and body weights were measured periodically to evaluate the therapeutic efficacy of the test agents (*n* = 5). (B) Tumor growth curves following various treatments. (C) Dynamic changes in body weight of mice after different treatments. (D) Survival curve of CT26 tumor-bearing mice. (E) Representative histological features and immunofluorescence staining (HK-2, DLAT, and TUNEL) of tumor tissues from CT26 tumor-bearing mice of different treatment groups. Scale bar, 50 μm. Quantification of fluorescence intensity of HK-2 (F), DLAT (G), and TUNEL (H). (I) WB analysis of HK-2 and DLAT. Quantitative analysis of HK-2 (J) and DLAT (K) was conducted by ImageJ. (L) WB analysis of FDX1 in tumor tissues. (M) Quantitative analysis of DLAT using ImageJ, and β-actin served as a loading control. For (B), statistical analysis was conducted using two-way ANOVA. For the survival curve comparison in (D), the log-rank (Mantel–Cox) test and Gehan–Breslow–Wilcoxon test were employed, and both revealed statistically significant differences in survival curves between groups (*P* < 0.05). Furthermore, the log-rank test for trend further indicated significant trend differences in survival outcomes across groups (*P* < 0.05). For all other quantitative data, statistical analysis was performed using one-way ANOVA.

After proving the excellent cytotoxicity of CHNDs in treating 4T1 orthotopic tumor, the potential feasibility of CHNDs in treating CT26 colon cancer xenograft model was further explored. Mice bearing established tumors were treated according to the protocol outlined in Fig. 8A. Mice bearing CT26 tumors were randomly assigned to 4 treatment groups: PBS, PHDs, CuDT, and CHNDs. Antitumor efficacy in the CT26 colon tumor model was evaluated by monitoring tumor growth curves and survival. As shown in Fig. 8B, all treatment groups exhibited significant inhibition of tumor growth compared with the PBS group. Both PHDs and CuDT treatments delayed tumor progression to some extent. As expected, the CHNDs treatment group exhibited the most pronounced suppression of tumor growth compared to the PBS group, with a statistically significant difference observed compared to the other 3 groups, with tumor inhibition rate to 76.6% (*P* < 0.0001). Notably, no significant changes in body weight were observed throughout the treatment period (Fig. 8C), indicating that the formulations caused minimal systemic toxicity. Survival analysis further underscored the therapeutic benefit of CHNDs. Compared to a median survival of 21 d for the PBS group, the CHNDs treatment resulted in strong tumor growth inhibition, with 20% of animals surviving at day 100 (Fig. 8D). Representative images of tumor-bearing mice visually corroborated these findings, demonstrating reduced tumor volumes in all treatment groups compared with the PBS group, with the smallest tumors observed in the CHNDs group (Fig. S24). Collectively, these data demonstrated the potent in vivo antitumor efficacy of CHNDs nanodrugs in the CT26 tumor model, highlighting their potential as a broad-spectrum anticancer therapeutic strategy.

The excellent therapeutic efficacy of CHNDs was further confirmed by histological and immunofluorescence staining and TUNEL analysis. As shown in Fig. 8E, H&E and TUNEL staining revealed that CHNDs-treated tumors exhibited extensive apoptosis and necrosis, which was more pronounced than in the PBS control and CuDT groups. To gain deep investigation on the antitumor mechanism of CHNDs in vivo, DLAT, FDX1, and HK-2 expressions in tumors were detected by immunofluorescence and WB assay. Immunofluorescence staining for HK-2 revealed a significant down-regulation in both the PHDs (*P* = 0.0002) and CHNDs groups (*P* = 0.0038) compared to the PBS group (Fig. 8E). Quantitative analysis showed that PHDs and CHNDs treatments reduced HK-2 protein levels in tumor tissues by 44.8% and 79.3%, respectively (Fig. 8F). In line with in vitro results, more pronounced DLAT aggregation, HK-2 degradation, and less FDX1 in the CHNDs treatment group were observed, verifying glycolysis regulation and the presence of cuproptosis in vivo (Fig. 8E and G to M). Quantitative analysis revealed that CHNDs treatment decreased DLAT and FDX1 levels by 41.2% and 60.8%, respectively (Fig. 8I to M). Notably, throughout the treatment period, the major organs (heart, liver, spleen, lung, and kidney) of mice in the CHNDs group exhibited no detectable histopathological lesions (Fig. S25), further indicating that CHNDs were well tolerated and demonstrated biosafety for synergistic cuproptosis and glycolysis-targeted anticancer therapy.

### Inhibition of lung metastasis

**Fig. 9.**
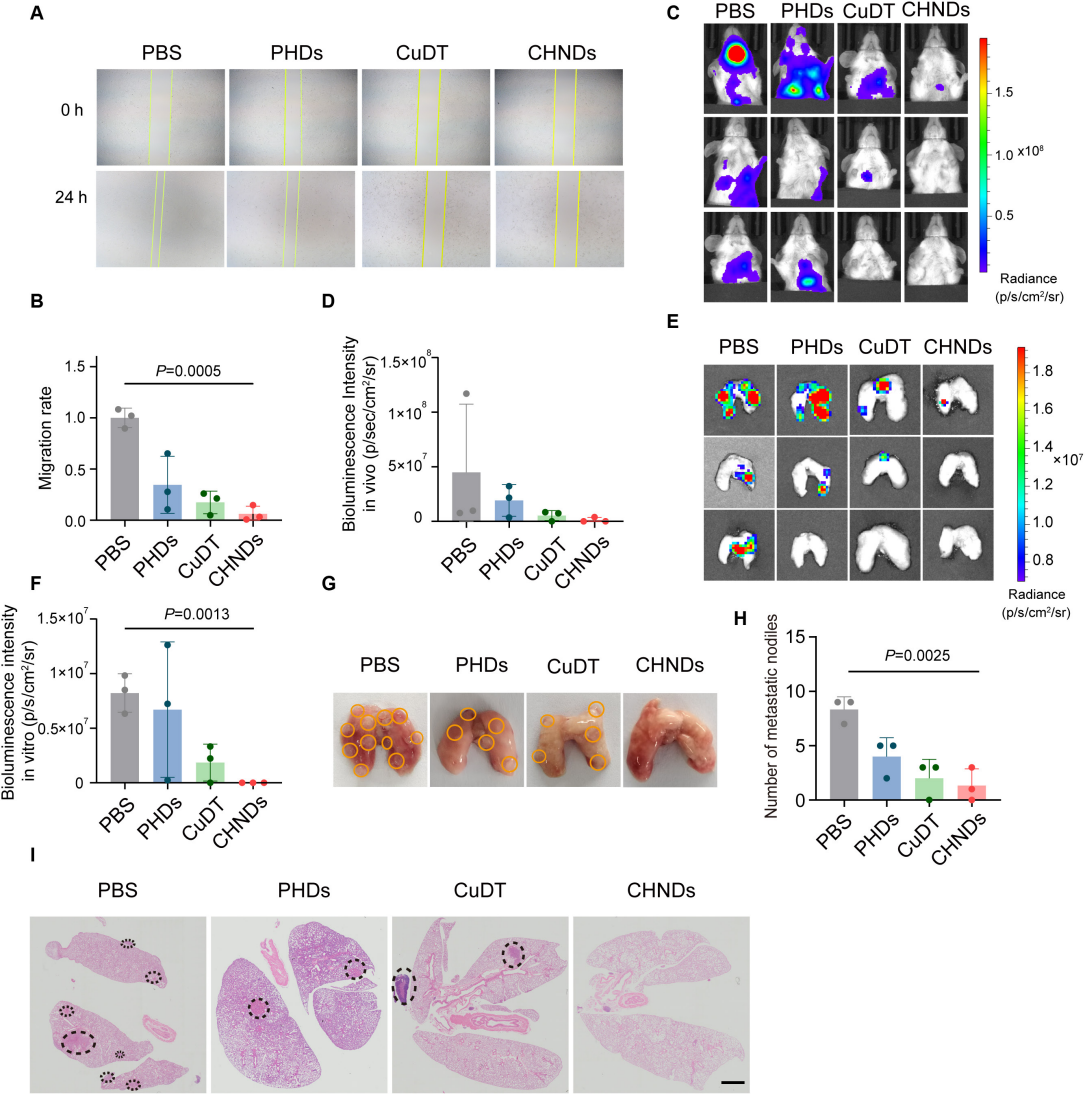
Inhibition of metastasis induced by CHNDs. (A) The wound-healing assay was employed to assess the capacity of CHNDs to inhibit cell migration. (B) Quantitative statistical results of cell mobility. Pulmonary metastatic nodule bioluminescence images (C) and quantitative analysis (D) in 4T1 tumor-bearing mice. The ex vivo bioluminescence images (E) and quantitative analysis (F) of metastatic nodules in the lungs. (G) Representative micrographs of lung tissues following different treatment regimens. (H) Quantitative analysis of metastatic nodules ex vivo in the lungs. (I) H&E staining of lung tissues of 4T1 tumor-bearing mice after treatment with indicated drugs. All significance analyses in this figure were performed using one-way ANOVA.

Given the potent synergistic antitumor activity of CHNDs, we further investigated their effect on suppressing lung metastasis in mice bearing orthotopic breast tumors. We first assessed the impact of CHNDs on tumor cell migration by in vitro scratch assay. As shown in Fig. 9A, untreated 4T1 cells exhibited robust migratory capacity, evidenced by significant wound closure after 24 h. In contrast, cells treated with CHNDs displayed minimal wound closure, indicating a substantial impairment of cell migration. Quantitative analysis of the wound area (Fig. 9B) revealed that PHDs, CuDT, and CHNDs treatments reduced migration rate to 34.5%, 17.4%, and 6.3% of the control, respectively. These data demonstrated the potent suppression of the migration and metastatic ability of tumors subjected to CHNDs treatment.

Furthermore, we employed a spontaneous pulmonary metastasis model using luciferase-expressed 4T1 breast tumor-bearing mice. In vivo bioluminescence imaging (Fig. 9C and D) revealed strong bioluminescence intensity of the lungs in the PBS-treated mice, indicative of extensive metastatic colonization. Occasional cranial bioluminescence signals were observed in PBS-treated mice, consistent with previously reported brain metastasis in the 4T1 model [50]. Treatment with either PHDs or CuDT resulted in weaker bioluminescence intensity of the lungs compared to the PBS group, suggesting a degree of metastasis suppression. In contrast, the CHNDs treatment group exhibited lower bioluminescence intensity than the other groups, demonstrating a potent inhibitory effect on lung metastasis ex vivo and in vivo (Fig. 9C to F). Additionally, CHNDs-treated mice exhibited fewer tumor nodules on the lung surface compared with PBS- or PHDs-treated mice (*P* = 0.0025) (Fig. 9G to I). These histological results further corroborated the above data and provided compelling visual evidence of the antimetastatic efficacy of CHNDs.

## Discussion

Cuproptosis has emerged as a promising antitumor strategy that induces copper-dependent mitochondrial proteotoxic stress; however, its therapeutic efficacy is frequently constrained by tumor metabolic reprogramming, particularly enhanced glycolysis, which mitigates copper-induced cytotoxicity [49,51]. Recent studies indicate that combining cuproptosis with complementary therapeutic modalities can enhance antitumor efficacy, highlighting the importance of synergistic metabolic interventions [52,53].

In this study, we demonstrated that integrating cuproptosis with HK-2 degradation effectively restricted glycolytic compensation and exacerbated mitochondrial dysfunction. By selectively targeting HK-2 (a central regulator of glycolysis and mitochondrial integrity), CHNDs simultaneously suppressed glycolytic flux and facilitated copper-induced metabolic collapse through stimulus-responsive, spatiotemporally controlled release.

Collectively, these findings identified targeted degradation of glycolytic enzymes as an effective strategy to potentiate cuproptosis and overcome tumor metabolic adaptability. Moreover, the CHNDs platform established a mechanistic framework for integrating PROTAC technology with metal-induced cell death, thereby offering a promising avenue for precision metabolic cancer therapy. Future studies should focus on elucidating the immunological effects of CHNDs-based therapeutic strategies, as well as systematically evaluating potential metal ion accumulation and associated toxicities in vivo.

## Materials and Methods

### Materials and agents

Hexafluorophosphate (HATU), 4-dimethylaminopyridine (DMAP), N, N diisopropylethylamine (DIPEA), and MTT were purchased from Sigma-Aldrich. 3-BP, 4-F-THA, polyethyleneimine (molecular weight = 1,800 Da; branched), and methoxyl PEGylated derivate (mPEG_2k_-OH) were purchased from Adamas Beta. Annexin V-FITC/PI Apoptosis Kit and JC-1 Mitochondrial Membrane Potential Kit were obtained from Beyotime (Shanghai, China). Rabbit anti-HK-2, anti-FDX1, and anti-DLAT antibodies, as well as mouse anti-β-actin antibody, were purchased from Proteintech (Wuhan, China).

### Cell lines and animals

4T1, CT26, and luciferase-expressing 4T1 cells were cultured in RPMI 1640 supplemented with 10% FBS at 37 °C under 5% CO_2_. Cells were harvested using trypsin containing 0.25% EDTA when the cell density reached above 80%. Specific pathogen-free (SPF) BALB/c mice (6 to 8 weeks old) were obtained from Vital River Laboratory Animal Center (Beijing, China) and housed in a standard facility with controlled temperature and humidity. All animal procedures were reviewed and approved by the Institutional Animal Ethics Committee of the Institute of Medical Research, Northwestern Polytechnical University, in accordance with relevant ethical guidelines.

### Synthesis of PHDs

Anhydrous *N*,*N*-dimethylformamide (DMF) (1.5 ml) was used to dissolve 40 mg of PEI, 6.14 mg of 4-F-THA, and 4 μl of DIPEA. The mixture was stirred at 90 °C overnight, after which 3.7 mg of 3-BP and 16.9 mg of HATU were added, and the resulting solution was stirred at 37 °C for 4 h. The crude THA–PEI–3-BP (PHDs) was purified sequentially by precipitation with ice-cold ether and dialysis in deionized water for 12 h using a 1,000-Da dialysis membrane. Finally, lyophilization afforded the pure product.

### Synthesis of PEG-PHDs

CH_3_O-PEG_2k_-OH (40 mg), succinic anhydride (6.0 mg), and DMAP (4.9 mg) were dissolved in 2.0 ml of anhydrous dichloromethane (DCM). The reaction mixture was stirred at ambient temperature overnight. The crude product, CH_3_O-PEG_2K_-SA, was purified by precipitation with ice-cold ether followed by drying. CH_3_O-PEG_2K_-SA (47.5 mg), PHDs (48.9 mg), DIPEA (4 μl), and HATU (16.8 mg) were dissolved in 2 ml of anhydrous DMF. The mixture was stirred for overnight at room temperature. The crude product, PEG-PHDs, was purified by precipitation with ice-cold ether followed by dialysis using 1**,**000-Da dialysis bag in ultra-pure water for 12 h. The pure product was obtained by lyophilization.

### Synthesis of Ce6-labeled PEG-PHDs

PEG-PHDs (10 mg), Ce6 (1.4 mg), DIPEA (2 μl), and HATU (1.8 mg) were dissolved in 2 ml of anhydrous DMF. The reaction mixture was stirred at ambient temperature overnight. The crude product, Ce6-labeled PEG-PHDs, was collected and dialyzed overnight with a dialysis bag [molecular weight cutoff (MWCO): 1,000 Da] in alcohol solution and then for 4 h in deionized water.

### Characterization of PEG-PHDs

^1^H NMR spectroscopy confirmed the structure of PEG-PHDs. ^1^H NMR of PEG-PHDs [500 MHz, dimethyl sulfoxide (DMSO)-d6, δppm] δ 7.28 (m, 3H), 5.76 (s, 1H), 4.11 (s, 1H), 3.51 (s, 181H), 3.24 (s, 3H), 2.82 (m, J = 79.9 Hz, 77H). FTIR spectroscopy was measured on an FTIR spectrophotometer (Thermo, USA). The purity of PHDs was detected by high-performance liquid chromatography (HPLC). Column: Agilent C18 reverse column (4.6 × 250 mm, 5 μm). Mobile phase: methanol:water = 70:30. Column temperature: 25 °C. Detection wavelength: 237 nm. Flow rate: 1 ml/min. Injection volume: 10 μl.

### Preparation and characterization of CHNDs

PVP K90 (16 mg) was first dissolved in 4 ml of deionized water and added into DTC·3H_2_O (1.8 mg) aqueous solution. CuCl_2_·2H_2_O (0.68 mg) was dissolved in 2 ml of deionized water. The solution of CuCl_2_ was slowly added into the mixture solution of PVP K90 and DTC under continuous stirring. After stirring at a constant speed for 30 min, the nanoparticles were collected via dialysis with 14-kDa dialysis bag in water for 12 h. In order to obtain CHNDs, 0.2 ml of PEG-PHDs (7.9 mg) of DMSO solution was slowly dropped into CuDT NP solution under stirring continuously. After stirring at a constant speed for 2 h, the CHNDs were collected via dialysis with 14-kDa dialysis bag in water for 12 h.

The morphology of CuDT and CHNDs NPs was characterized by TEM. The size distribution of CuDT, non-PEG CHNDs, and CHNDs was detected by DLS. UV absorption spectrum and quantitative analysis was conducted by UV–Vis spectrophotometer.

### Hydroxyl radical (·OH) detection by MB assay

·OH generation via the copper ion-mediated Fenton-like reaction was investigated by MB as a probe. Different concentrations of CHND suspensions were added into PBS (pH 6.8) containing hydrogen peroxide (H₂O₂, 100 nM) and MB (5 μg/ml). A control group without H₂O₂ solution was concurrently prepared to eliminate the influence of intrinsic solution color on absorbance readings. The absorption spectrum of the solution was performed by UV–Vis spectroscopy over the wavelength range of 400 to 800 nm.

### Drug release study

For drug release study, CHNDs suspension was placed into a dialysis bag (MWCO: 14,000 Da), and the bag was immersed in a 3.8-ml pH 7.4 PBS solution and a 3.8-ml pH 5.0 PBS solution containing 10 mM GSH in a shaker (37 °C, 100 rpm). At specified time points (0, 0.25, 0.5, 1, 2, 4, 6, 12, 24, and 48 h), 0.2 ml of PBS medium was collected for analysis and replaced with an equal volume of fresh medium. The amount of PEG-PHDs was measured by a UV–Vis spectroscopy.

### GSH depletion in vitro

Different concentrations of CHNDs suspension (0, 10, 20, and 50 μg/ml) were mixed with pH 5.0 PBS solution with 10 mM GSH for 30 min at 37 °C. 5,5′-Disulfide (2-nitrobenzoic acid) (DTNB) solution (2.5 mg/ml) was then added to the above solution, followed by continuous stirring for 5 min. After that, the absorbance at 412 nm was measured by the UV–Vis spectroscopy.

### WB assay

CHNDs-mediated degradation of HK-2 and cuproptosis-associated proteins (e.g., DLAT and FDX1) was assessed by WB. Briefly, 4T1 and CT26 cells were seeded into 6-well plates at a density of 5 × 10^5^ cells per well. After overnight incubation at 37 °C with 5% CO_2_, the culture medium was replaced with PHDs, CuDT, or CHNDs under pre-established conditions. At the end of the experiment, cell lysates were collected, and protein concentrations were determined using the bicinchoninic acid (BCA) assay. Following normalization, protein samples were denatured at 95 °C for 10 min. Equal amounts of protein were separated on 8% to 15% sodium dodecyl sulfate–polyacrylamide gel electrophoresis (SDS-PAGE) gels and transferred onto polyvinylidene fluoride (PVDF) membranes (Millipore). After blocking with 5% nonfat dry milk for 1 h at room temperature, membranes were washed 3 times with 1× tris-buffered saline–Tween 20 (TBST). Membranes were then incubated with primary antibodies overnight at 4 °C, followed by 3 washes with TBST for 10 min. Subsequently, membranes were incubated with horseradish peroxidase (HRP)-conjugated secondary antibodies for 2 h at room temperature. After 3 additional TBST washes, membranes were treated with a chemiluminescent HRP substrate, and images were acquired using the FUSION FX6 EDGE Imaging System (VILBER). Gray intensity of the resulting immunoblots was quantified using ImageJ software.

### Intracellular localization of CHNDs

4T1 cells were seeded in confocal dishes at a density of 5 × 10^3^ cells per well and cultured at 37 °C for 12 h. The cells were treated with Ce6-labeled CHNDs (Ce6: 20 μg/ml) for 3 h in a 5% CO_2_ incubator at 37 °C. Subsequently, MitoTracker Green was diluted in culture medium to a final concentration of 50 nM and added to the culture dishes, followed by incubation for 30 min to achieve sufficient mitochondrial staining. The cells were then further incubated with Hoechst 33342 (5 μg/ml) for 15 min in a humidified incubator (5% CO_2_) at 37 °C. After 3 washes with ice-cold PBS, fluorescence images showing the colocalization of nanoparticles with mitochondria were acquired using confocal microscopy.

### HK-2 degradation in vitro

4T1 and CT26 cells were seeded into separate 6-well plates and cultured for 24 h. When cell confluence reached approximately 50%, various formulations were diluted to predetermined concentrations in serum-free RPMI 1640 medium, added to the wells, and incubated for 3, 6, 9, 12, or 24 h. Cells were subsequently lysed to extract total protein, and HK-2 expression was analyzed by WB.

4T1 and CT26 cells were inoculated into 6-well plates for 24 h. When the cell confluence reached 50%, different formulations were diluted to 0.1, 1, 10, 20, and 40 μg/ml with serum-free RPMI 1640 medium added to the 6-well plate and incubated for 9 h. The cell protein was extracted, and the content of HK-2 protein was detected by WB assay.

To investigate the mechanism underlying CHNDs-mediated HK-2 degradation, 4T1 cells were seeded into 12-well plates and cultured for 24 h. Cells were treated with CHNDs alone or CHNDs in combination with MG132 (100 nM) or MLN4924 (100 nM), with each group receiving an equivalent 3-BP dose of 5 μg/ml, and then incubated for an additional 24 h. After 3 washes with cold PBS, cells were harvested and HK-2 protein levels were analyzed by WB.

### ROS generation

4T1 cells were seeded into 12-well plates at a density of 1 × 10^5^ cells per well and cultured until confluence reached approximately 70%. Cells were then treated with various formulations (PHDs, CuDT, and CHNDs) for 12 h in a humidified incubator at 37 °C with 5% CO_2_. The drug was removed and replaced with DCFH-DA (10 μM), incubating with cells for 20 min at 37 °C. The cells were washed with cold PBS for 3 times and collected to detect the fluorescence signals of DCF in different treatments.

### Glycolysis inhibition

Real-time ECAR was measured using a Seahorse XFe-96 analyzer (Agilent Technologies, USA). 4T1 cells (1.5 × 10^4^ cells per well) were seeded into Seahorse XFe-96 plates and cultured with various formulations for 24 h. Prior to measurement, the culture medium was replaced with Seahorse XF Base Medium (Agilent Technologies, USA) supplemented with glucose (10 mM), oligomycin (2 μM), and 2-deoxyglucose (50 mM), which were sequentially injected at the indicated time points. At the experiment endpoint, the cell culture plate was loaded into the analyzer and ECAR was recorded automatically by the Seahorse XFe-96 analyzer. Data were analyzed by GraphPad Prism software.

### Cytotoxicity in vitro

The in vitro cytotoxicity was assessed using the MTT assay. 4T1 cells were seeded into 96-well plates at a density of 5,000 cells per well and cultured under standard conditions (37 °C, 5% CO_2_) for 12 h. The culture medium was then replaced with fresh medium containing varying concentrations of 3-BP, DTC, PHDs, CuDT, and CHNDs. After 48 h of incubation, MTT solution (5 mg/ml) was added to each well and incubated for an additional 4 h. The MTT-containing medium was subsequently removed, and an appropriate volume of DMSO was added to each well to dissolve the formazan crystals. Finally, a microplate reader was used to investigate the relative cell viabilities at an absorbance of 490 nm.

Following treatment with different groups, cytotoxicity was also carried out by calcein-AM/PI (live/dead cell) staining assay. 4T1 cells were seeded into a 6-well cell culture plate, followed by PHDs, CuDT, and CHNDs treatment for 24 h. Afterward, calcein-AM (5 μg/ml) and PI (10 μg/ml) were used to stain the 4T1 cells. The images were observed by an Inverted Fluorescence Microscope Spectrophotometer (OLYMPUS IX73).

To assess mitochondrial membrane potential, 4T1 cells were treated with various formulations (PHDs, CuDT, and CHNDs) for 24 h, followed by JC-1 staining and fluorescence imaging using a confocal laser scanning microscope.

### Apoptosis assay

Cellular apoptosis was evaluated using an Annexin V-FITC/PI apoptosis detection kit. Briefly, 4T1 cells were seeded into 6-well plates at a density of 1 × 10^6^ cells per well and cultured overnight. The cells were then treated with PBS, PHDs, CuDT, or CHNDs for 24 h. After staining with the Annexin V-FITC/PI kit according to the manufacturer’s instructions, the percentage of apoptotic cells were quantified by flow cytometry.

### Colony formation assay

4T1 cells were seeded into 6-well plates at a density of 1,000 cells per well and incubated overnight under standard culture conditions (37 °C, 5% CO_2_). The cells were then treated with various formulations for 7 d. After 3 washes with cold PBS, the cells were fixed with 4% paraformaldehyde and stained with crystal violet for 15 min. Following washing with ultrapure water, the plates were air-dried at room temperature and imaged using a digital camera.

### Immunofluorescence assay

4T1 cells (5 × 10^4^ cells per well) were seeded into glass-bottom confocal dishes, cultured for 12 h, and treated with PHDs, CuDT, or CHNDs for 24 h. Cells were subsequently fixed with 4% paraformaldehyde (room temperature, 20 min), permeabilized with 0.2% Triton X-100, blocked with 5% bovine serum albumin (BSA), incubated with the primary antibody at 4 °C overnight and the secondary antibody at room temperature for 2 h, counterstained with Hoechst 33342 for 10 min, and imaged using confocal microscopy.

### WB analysis of DLAT, FDX1, and LIAS

The cells were inoculated into a 6-well plate, cultured overnight in a cell incubator at 37 °C with 5% CO_2_. After that, 4T1 cells were divided into 4 groups for WB assay: (a) PBS, (b) PHDs, (c) CuDT, and (d) CHNDs with equal concentration of 3-BP (2 μg/ml) and CuDT (4.5 μg/ml). After being incubated for 24 h, the content of FDX1, DLAT, and LIAS was detected by WB experiment according to the protocol. β-Actin was employed as protein loading control.

### Intracellular ATP content detection

4T1 cells were seeded into 12-well plates at a density of 1 × 10^5^ cells per well for 24 h. The cells were subsequently treated with different preparations (PBS, PHDs, CuDT, or CHNDs) for 12 h, followed by digestion and collection via centrifugation. Intracellular ATP levels were then measured using an ATP assay kit.

### Measurement of l-lactic acid content

4T1 cells were seeded into 12-well plates at a density of 1 × 10^5^ cells per well. After 24 h of culture, the cells were treated with PHDs, CuDT, or CHNDs for 12 h, and the extracellular l-lactic acid was subsequently measured using an l-lactic acid detection kit.

### Antitumor effect study in vivo

BALB/c mice were implanted with 2 × 10^5^ luciferase-expressing 4T1 cells via subcutaneous injection into the left fourth mammary fat pad to establish a breast tumor model. When tumors reached 50 to 100 mm^3^, the tumor-bearing mice were randomly assigned to 4 experimental groups and treated with PBS, PHDs, CuDT, or CHNDs every 2 d, with each treatment containing equivalent doses of 3-BP (2 mg/kg) and CuDT (4.5 mg/kg). Tumor volume and body weight were recorded every other day. The same treatment regimen was applied to CT26 subcutaneous tumor models. Mice were euthanized when tumor volume reached 1,500 mm^3^. Then, tumors and major organs were collected for pathological analyses, including WB, immunofluorescence staining, and H&E staining.

Blood samples were collected from 3 randomly selected mice per group 1 d after the final administration to assess biochemical parameters as part of biosafety evaluation. Tumor tissues were also harvested for detection of HK-2, FDX1, and DLAT protein levels using WB and immunofluorescence assays.

### Hemolysis study

A hemolysis assay was performed to evaluate the blood compatibility of CHNDs in vitro. Briefly, blood was collected from mice and centrifuged at 3,000 rpm for 10 min at 4 °C, and the supernatant was discarded to isolate red blood cells (RBCs). The RBCs were washed 3 times with cold PBS and adjusted to a 2.5% (v/v) suspension. The cells were then incubated with various formulations for 3 h, with distilled water serving as the positive control to validate assay specificity. Hemolysis rates were determined by measuring the absorbance of the solutions at 540 nm using a microplate reader.

### Wound healing

4T1 cells were seeded into 6-well plates at an optimal density and cultured until reaching approximately 70% confluency. A sterile 20-μl pipette tip was used to create a scratch in the cell monolayer, followed by 3 washes with ice-cold PBS to remove detached cells. Cells were then treated with PHDs, CuDT, or CHNDs. Cell migration into the scratch area was monitored using an inverted fluorescence microscope after 24 h of culture, and the wound-healing rate was quantified through image analysis.

### Anti-lung metastasis in vivo

4T1-Luc tumor-bearing mice were used as a model of spontaneous breast cancer metastasis, and a corresponding orthotopic 4T1-Luc tumor model was subsequently established. Tumor-bearing BALB/c mice were randomly assigned to 4 experimental groups and injected intravenously with PHDs, CuDT, or CHNDs (3-BP: 2 mg/kg, CuDT: 4.5 mg/kg) for 5 cycles at 1-d intervals. At the end of the study, each mouse received an intraperitoneal injection of 200 μl of d-luciferin potassium salt (15 mg/ml). After 8 min of incubation to allow signal maturation, bioluminescence imaging was performed using a PerkinElmer IVIS Lumina LT Series III in vivo imaging system. The lungs were then harvested and incubated in d-luciferin solution for 8 min, and bioluminescence signals were detected using IVIS.

### Statistical analysis

All quantitative data are presented as mean ± standard deviation (SD) and were obtained from at least 3 independent experiments unless otherwise stated. Statistical analyses were performed using Student’s 2-tailed *t* test, one-way analysis of variance (ANOVA), or two-way ANOVA, as appropriate for the experimental design. Specifically, Student’s *t* test was used for comparisons between 2 independent groups, while one-way ANOVA was applied for comparisons among 3 or more independent groups under a single experimental condition. Two-way ANOVA was employed for tumor growth curve analyses, with treatment group and time as independent factors, allowing evaluation of both main effects and their interaction. Prior to ANOVA, data were assessed for compliance with the assumptions of parametric testing, including approximate normality and homogeneity of variance. When a statistically significant difference was detected by ANOVA, Tukey’s post hoc test was performed for multiple comparisons between groups. Post hoc analyses were conducted only when the overall ANOVA result was significant. A *P* value of <0.05 was considered statistically significant. *P ≤ 0.05, **P ≤ 0.01, ***P ≤ 0.001.

## Data Availability

Data are available on reasonable request from the corresponding author.
