# Supplementary material for: Multifunctional Engineered Metal–Organic Frameworks as Targeted Protein Degraders for Augmenting Cancer Therapy via Hexokinase 2 Degradation and Provoking Cuproptosis
Source: Research (Wash D C). 2026 Mar 31;9:1217. doi: 10.34133/research.1217 (PMC13036323; doi:10.34133/research.1217)
Supplement: Supplementary 1 — Figs. S1 to S25 Tables S1 and S2 [file research.1217.f1.docx]

**Supporting Information**

**Multifunctional Engineered Metal Organic Frameworks as Targeted Protein Degraders for Augmenting Cancer Therapy via Hexokinase 2 Degradation and Provoking Cuproptosis**

Shasha Li^a, b^ , Runjie Liu^a, b^, Qixuan Zhang^a, b^, Jiahui Sun^a, b^, Hao Hao^b^, Chenhao Yao^c^, Li Yan^b^, Dan Yang^c^, Dechun Liu^a, b^ *

^a^ Research & Development Institute of Northwestern Polytechnical University in Shenzhen, Guangdong, 518057, China.

^b^ School of Life Science and Technology, Northwestern Polytechnical University, Xi’an, Shaanxi 710072, China

^c^ Department of Pharmaceutical Sciences, School of Biological and Pharmaceutical Sciences, Shaanxi University of Science and Technology, Weiyang University Park, Xi'an 710021, China

* Corresponding author

E-mail: [dechun.liu@nwpu.edu.cn](mailto:dechun.liu@nwpu.edu.cn) (D.C. Liu)

Table of Contents
Supplementary Fig.s
Fig. S1. The FTIR spectrum spectra of 3-BP, THA, PHDs and PEG-PHDs.

Fig. S2. Western blot analysis of HK-2 expression in CT26 cells treated with PHDs for different times.

Fig. S3. Western blot analysis of HK-2 expression in CT26 cells treated with different concentrations of PHDs.

Fig. S4. Western blot analysis of HK-2 degradation mechanism.

Fig. S5. The X-ray diffraction (XRD) pattern of CuDT and CHNDs NPs.

Fig. S6. XPS spectrum of non-PEG CHNDs.

Fig. S7. The N_2_ adsorption desorption and pore size distribution curves of CHNDs.

Fig. S8. The size stability and PDI of CHNDs.

Fig. S9. EDS mapping obtained by TEM for CHNDs.

Fig. S10. WB analysis of HK-2 expression in 4T1 cells treated with PHDs@CuDT for different times.

Fig. S11. WB analysis of HK-2 expression in CT26 cells treated with PHDs@CuDT for different times.

Fig. S12. WB analysis of HK-2 expression in CT26 cells treated with different concentrations of PHDs@CuDT.

Fig. S13. WB analysis of HK-2 expression in 4T1 cells treated with PHDs or PEG-PHDs for 12 h.

Fig. S14. The quantification of glycolytic capacity in 4T1 cells.

Fig. S15. Pearson’s correlation coefficient value and Mander’s colocalization coefficient value.

Fig. S16. Quantification of 4T1 cells colony formation assay.

Fig. S17. Representative fluorescence images of the intracellular ROS.

Fig. S18. Confocal microscopy images of JC-1 staining.

Fig. S19. Representative CLSM images of DLAT immunostaining.

Fig. S20. Representative pictures of luciferase-expressed 4T1 tumor bearing mice after different treatments.

Fig. S21. Blood routine examination.

Fig. S22. H&E staining of major organs (heart, liver, spleen, lung, and kidney) in 4T1 tumor bearing mice.

Fig. S23. *In vitro* hemolysis rate assay.

Fig. S24. Representative images of CT26 tumor bearing mice following different treatments.

Fig. S25. H&E staining of major organs (heart, liver, spleen, lung, and kidney) in CT26 tumor bearing mice.

**Table S1.** DLS date of CuDT and CHNDs

| Samples | Particle size (nm) | PDI | Zeta Potential (mV) |
| --- | --- | --- | --- |
| CuDT | 142.2±8.26 | 0.217±0.01 | -4.97±2.89 |
| CHNDs | 158.4±6.9 | 0.270±0.01 | -5.08±3.83 |

**Table S2.** IC_50_ value of 3BP, PHDs, CuDT, CHNDs in 4T1 cells

|  | IC_50_ (μg/mL) |
| --- | --- |
| 3-BP | - |
| PHDs | - |
| CuDT | 2.685 |
| CHNDs | 1.338 |


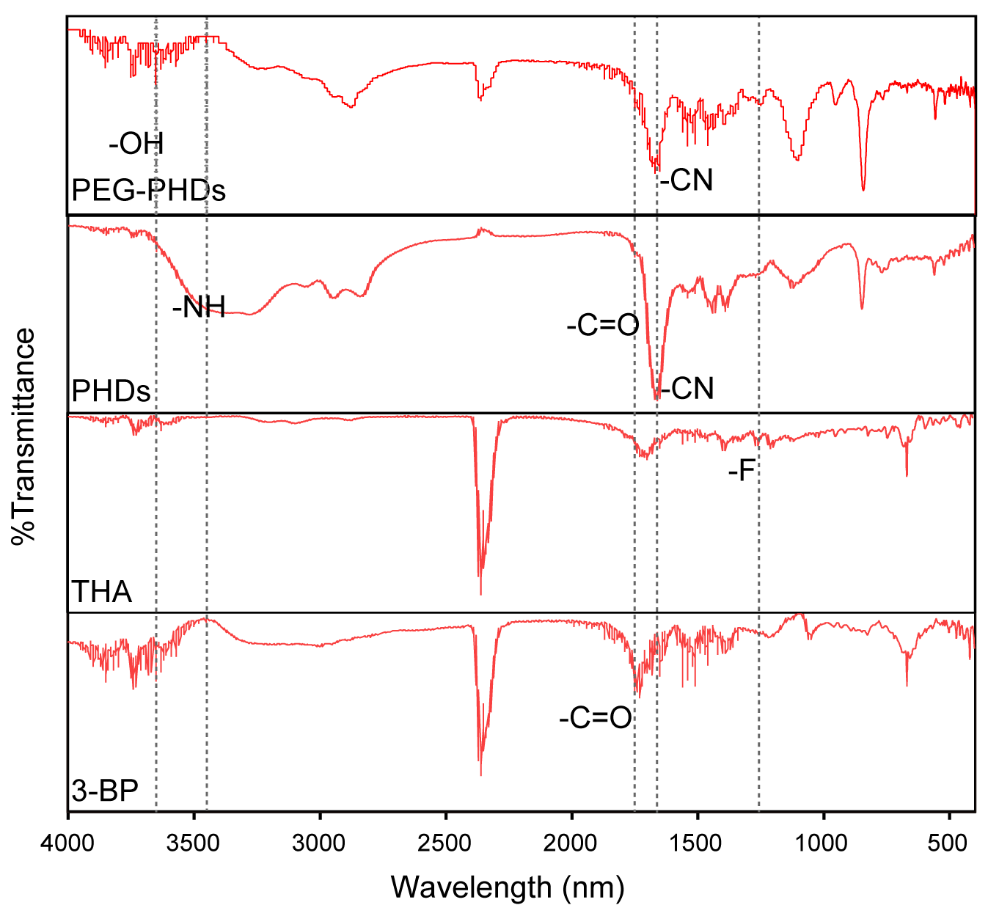


**Fig. S1.** The FTIR spectrum spectra of 3-BP, THA, PHDs and PEG-PHDs.


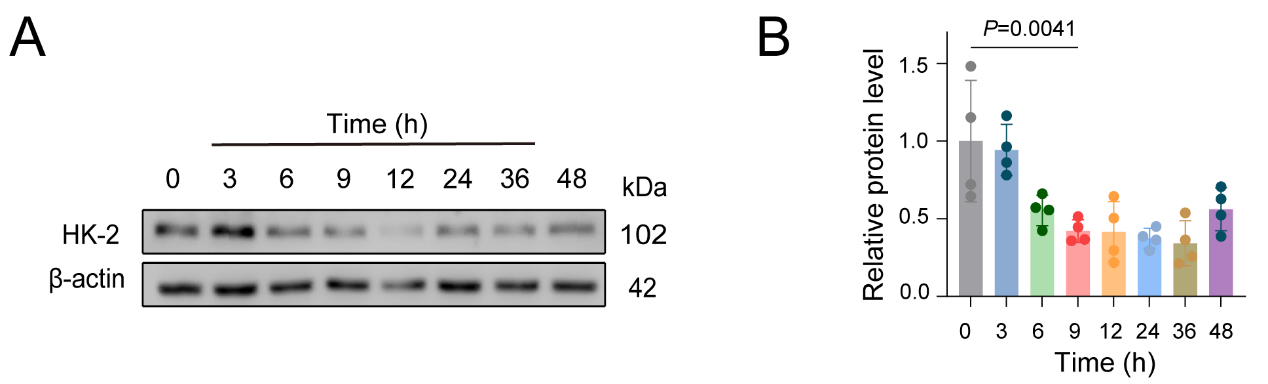


**Fig. S2. (A)** Western blot analysis of HK-2 expression in CT26 cells treated with PHDs (PHDs:10 μg/mL) for different times at 37 ℃. **(B)** Quantification of **(A)** by image J and β-actin was used for control.


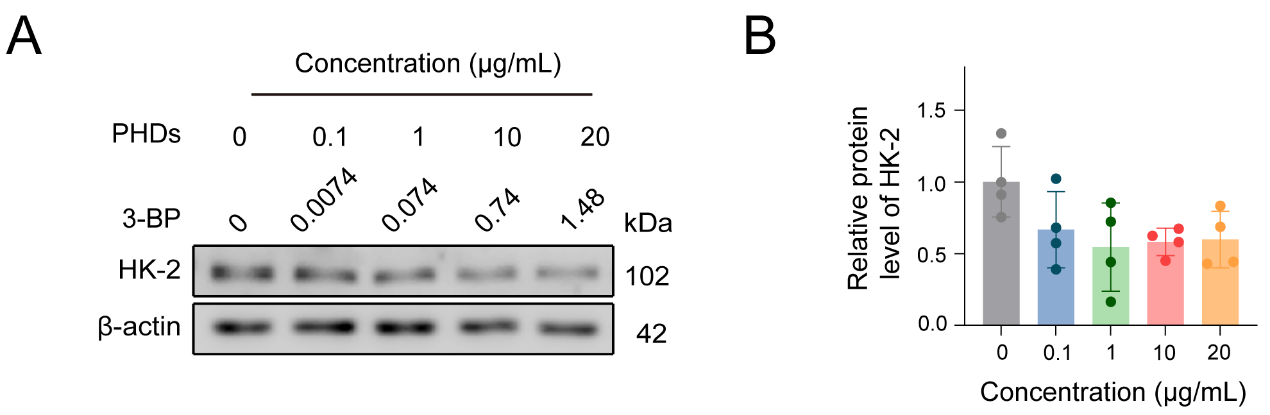


**Fig. S3. (A)** Western blot analysis of HK-2 expression in CT26 cells treated with PHDs for 9 h with different concentrations at 37 ℃. **(B)** Quantification of **(A)** by image J and β-actin was used for control.


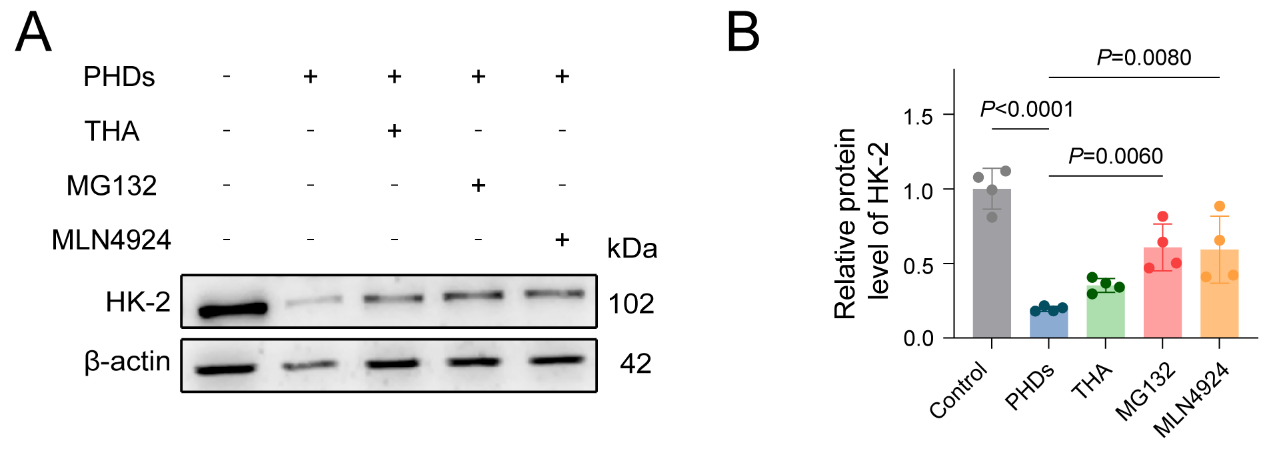


**Fig. S4. (A, B)** Western blot analysis and quantification of HK-2 expression in CT26 cells after co-cultured PHDs with THA, MG132, MLN4924 for 24 h. CT26 cells were pre-incubated with THA (20 μg/mL), MG132 (100 nM) or MLN4924 (100 nM) for 30 min, and then treated with PHDs (3BP: 2 μg/mL) for 24 h.


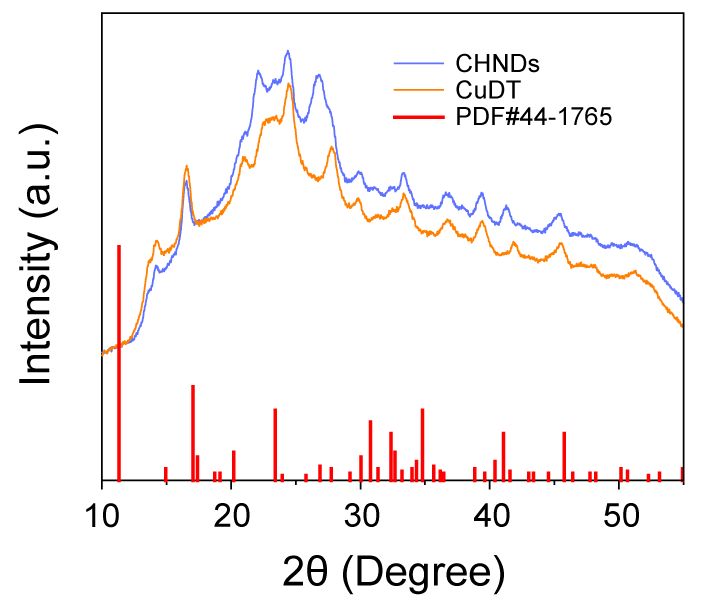


**Fig. S5.** The X-ray diffraction (XRD) pattern of CuDT and CHNDs NPs.


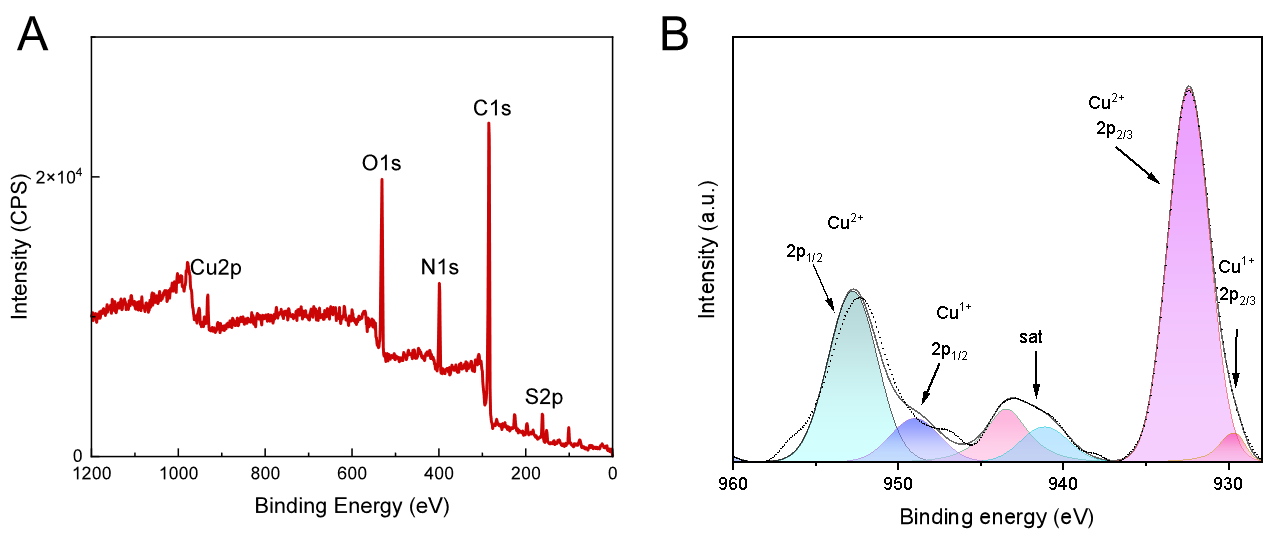


**Fig. S6.** (A) XPS spectrum of non-PEG CHNDs. (B) Cu 2p spectrum of non-PEG CHNDs.


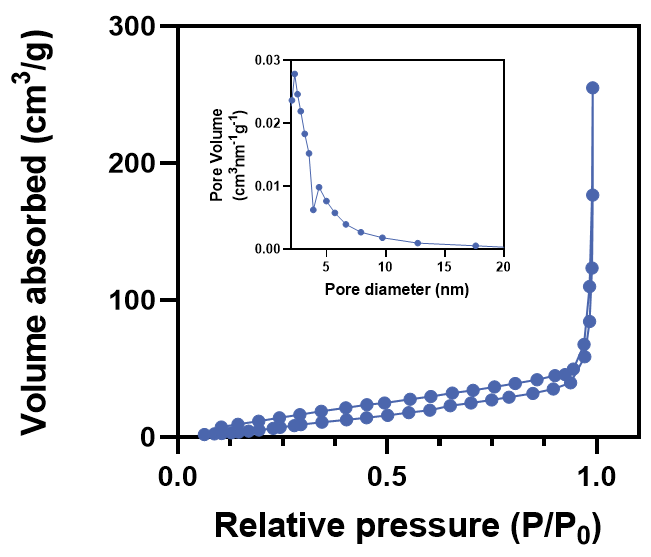


**Fig. S7.** The N_2_ adsorption desorption and pore size distribution curves of CHNDs.


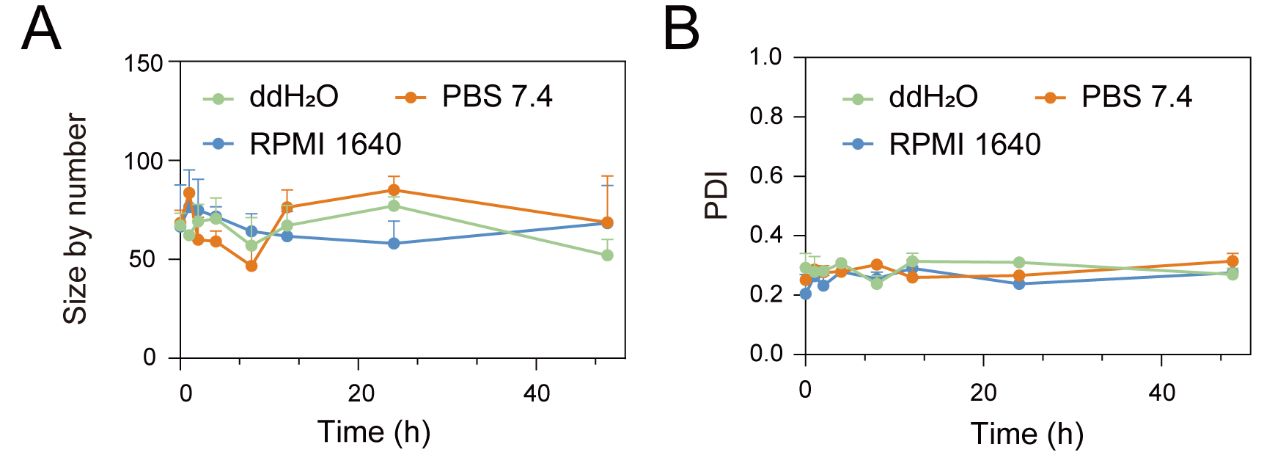


**Fig. S8. (A, B)** The size stability and PDI of CHNDs in deionized water (ddH_2_O), pH 7.4 PBS, RPMI 1640 containing 10% (V/V) FBS for 48 h detected by DLS.


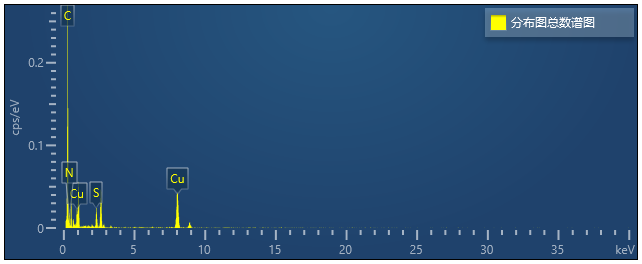


**Fig. S9.** EDS mapping obtained by TEM for CHNDs.


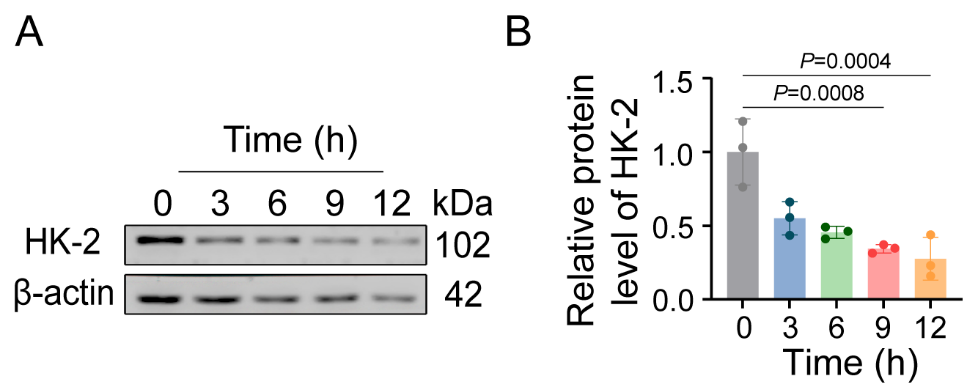


**Fig. S10. (A)** WB analysis of HK-2 expression in 4T1 cells treated with PHDs@CuDT (3-BP:1 μg/mL) for different times at 37 ℃. **(B)** Quantification of **(A)** by image J and β-actin was used for control.


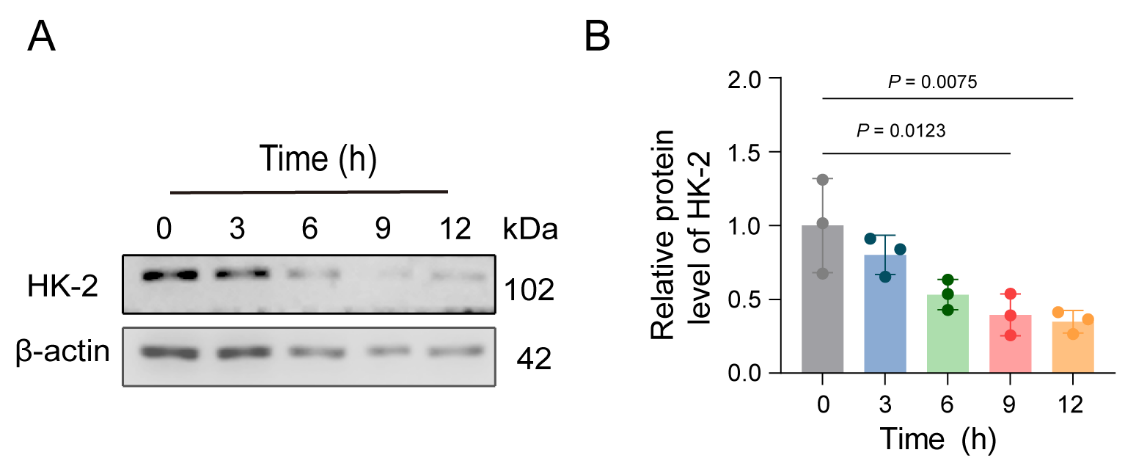


**Fig. S11. (A)** WB analysis of HK-2 expression in CT26 cells treated with PHDs@CuDT (3-BP:1 μg/mL) for different times at 37 ℃. **(B)** Quantification of **(A)** by image J and β-actin was used for control.


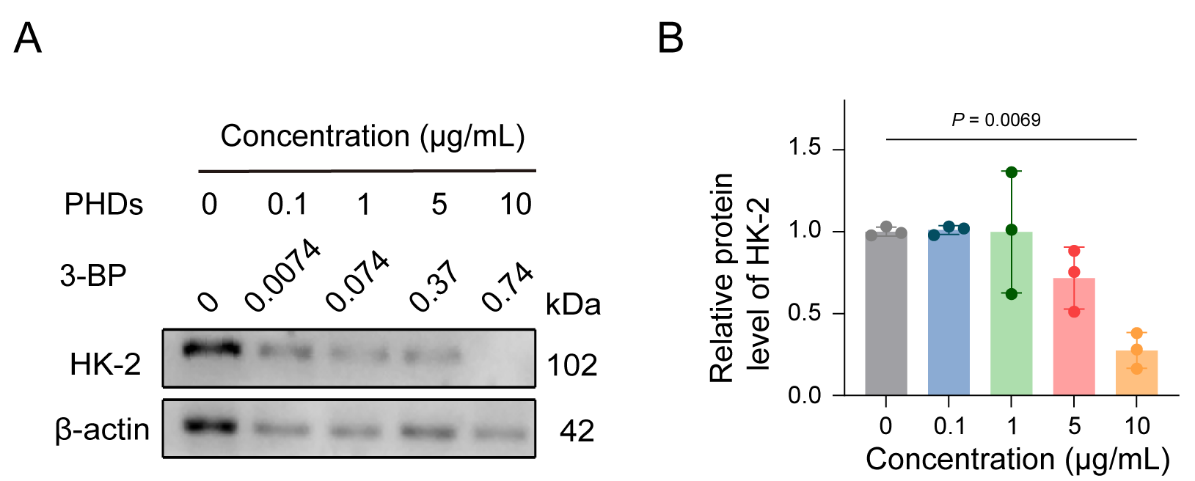


**Fig. S12. (A)** WB analysis of HK-2 expression in CT26 cells treated with PHDs@CuDT for 12 h with different concentrations at 37 ℃. **(B)** Quantification of **(A)** by image J and β-actin was used for control.


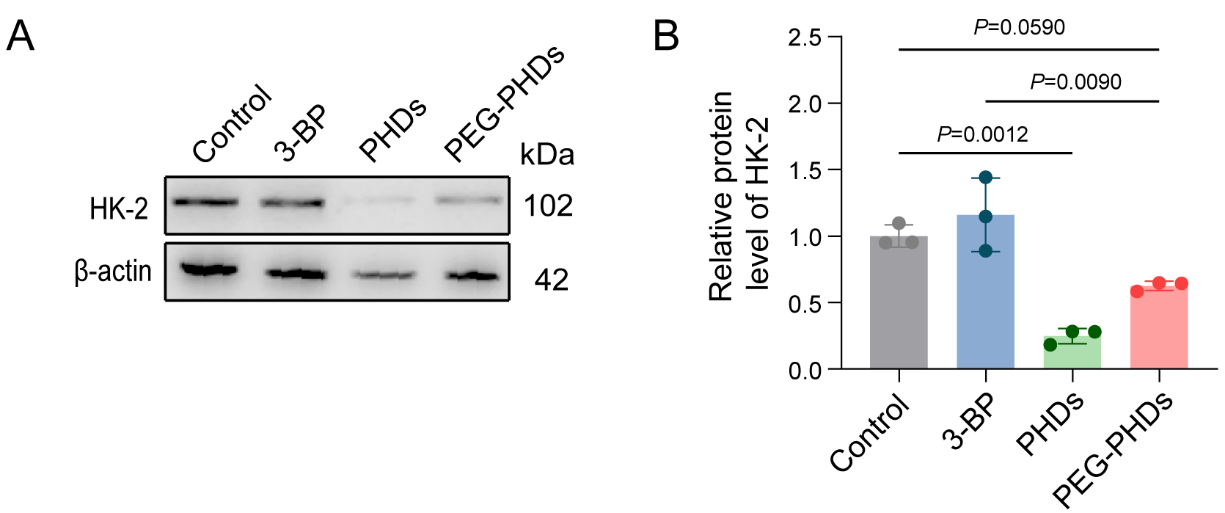


**Fig. S13.** **(A)** WB analysis of HK-2 expression in 4T1 cells treated with PHDs or PEG-PHDs for 12 h (3-BP:2 μg/mL). **(B)** Quantification of **(A)** by image J and β-actin was used for control.


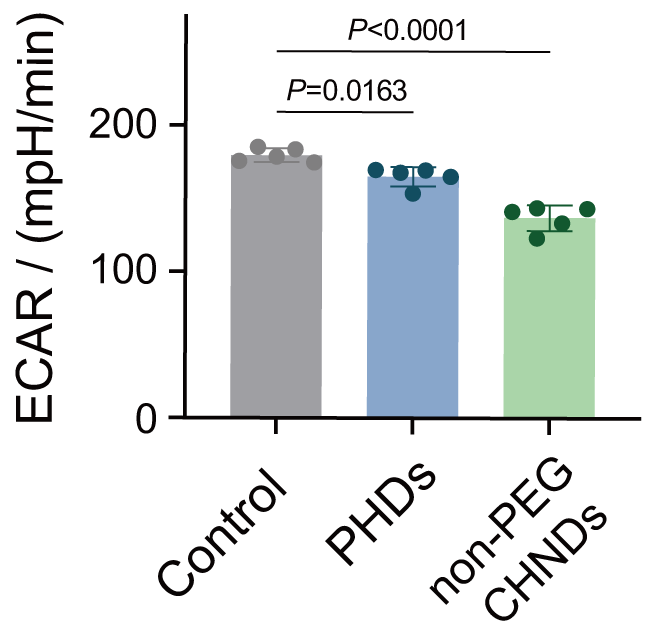


**Fig. S14.** 4T1 cells were incubated with PBS, PHDs, or PHDs@CuDT, and glycolytic capacity was subsequently quantified.


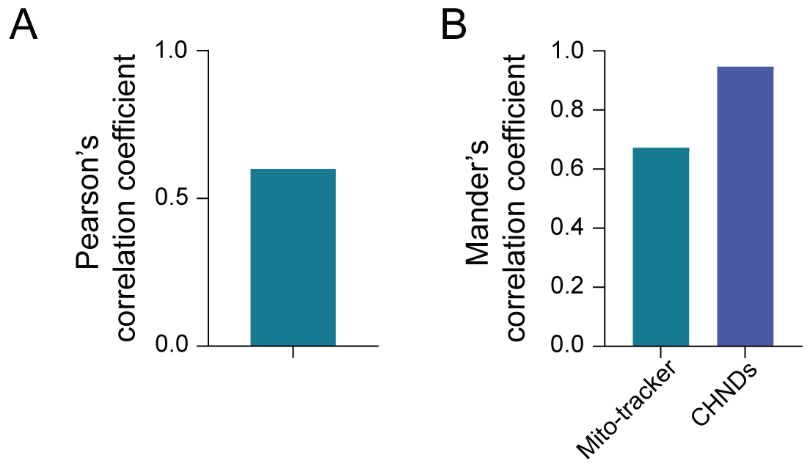


**Fig. S15.** Pearson’s correlation coefficient value **(A)** and Mander’s colocalization coefficient value **(B)** of colocalization between Mito-tracker and Ce6-labeled CHNDs and dates were analyzed by Image J.


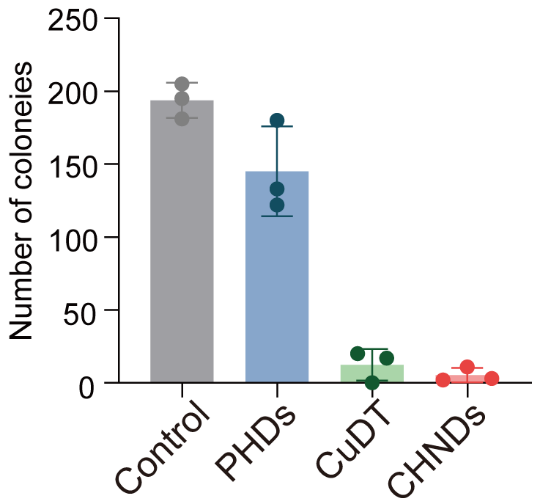


**Fig. S16.** Quantification of 4T1 cells colony formation assay after treated with PHDs, CuDT and CHNDs.


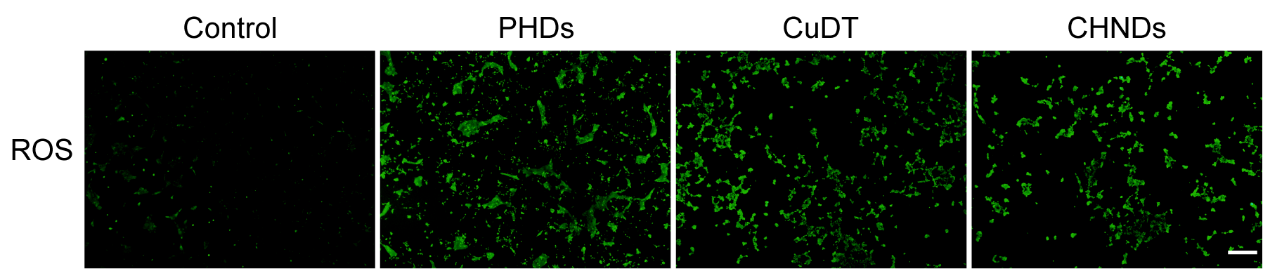


**Fig. S17.** Representative fluorescence images of the intracellular ROS (scale bar: 500 µm), and DCFH-DA was used as a probe to detect the intracellular ROS.


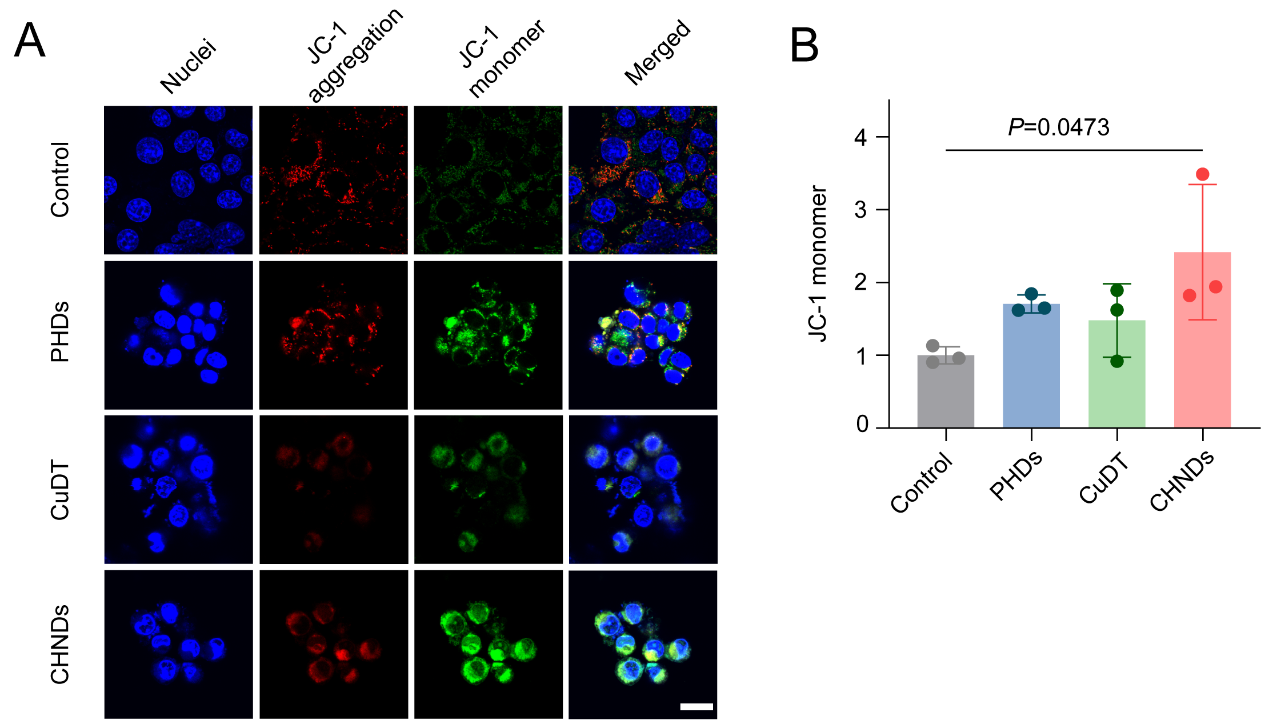


**Fig. S18. (A)** Confocal microscopy images of JC-1 staining in 4T1 cells treated with different treatments (3BP: 5 μg/mL) for 24 h to assess its impact on mitochondria membrane potential (Red: JC-1 aggregation, Green: JC-1 monomer, scale bar: 20 μm). **(B)** The quantification of JC-1 monomer by Image J.


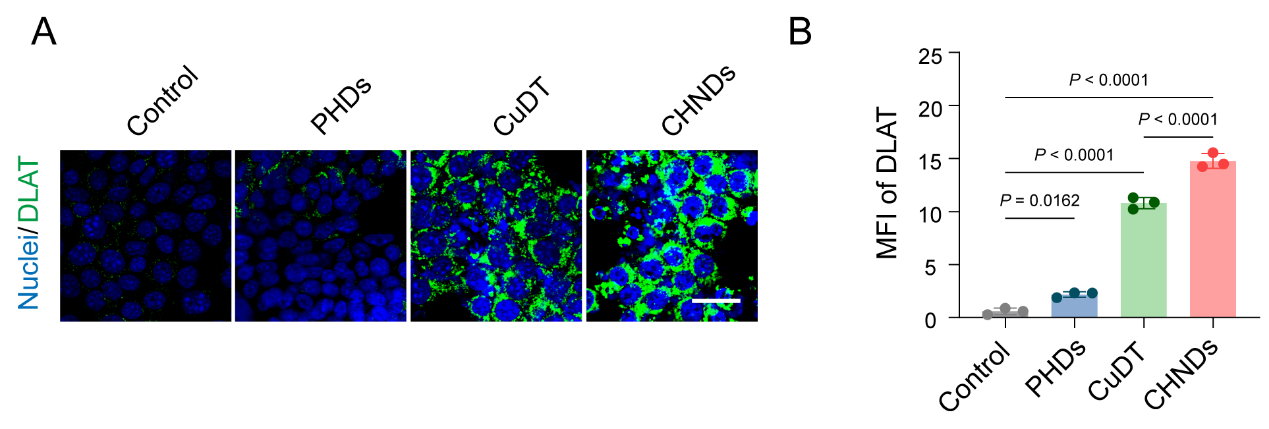


**Fig. S19.** Representative CLSM images of DLAT immunostaining **(A)** of 4T1 cells from after indicated treatments. Scale bar, 20 μm. **(B)** Quantification of **(A)** by Image J.


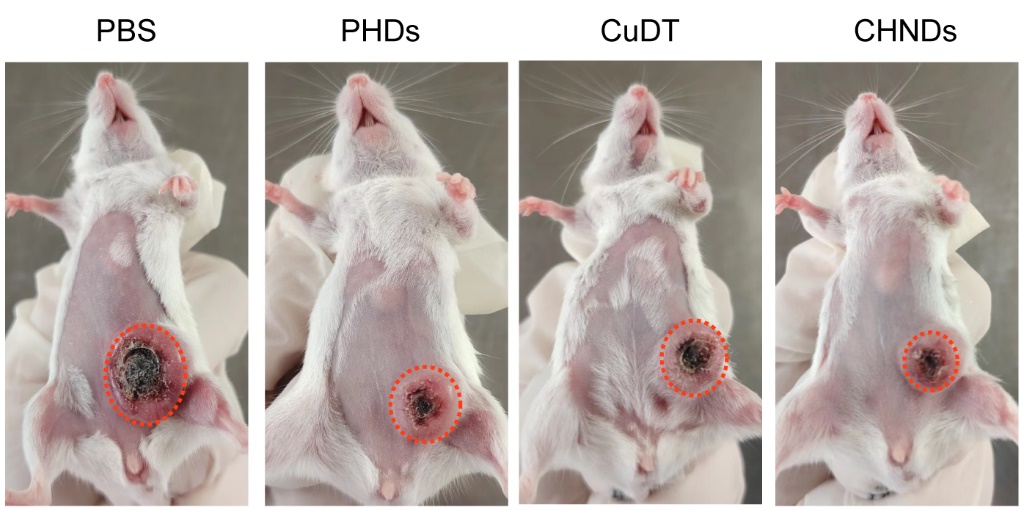


**Fig. S20.** Representative pictures of luciferase-expressed 4T1 tumor bearing mice after treated with indicated treatments (2 mg 3-BP/kg and 4.5 mg CuDT/kg, i.v.) for 5 times.


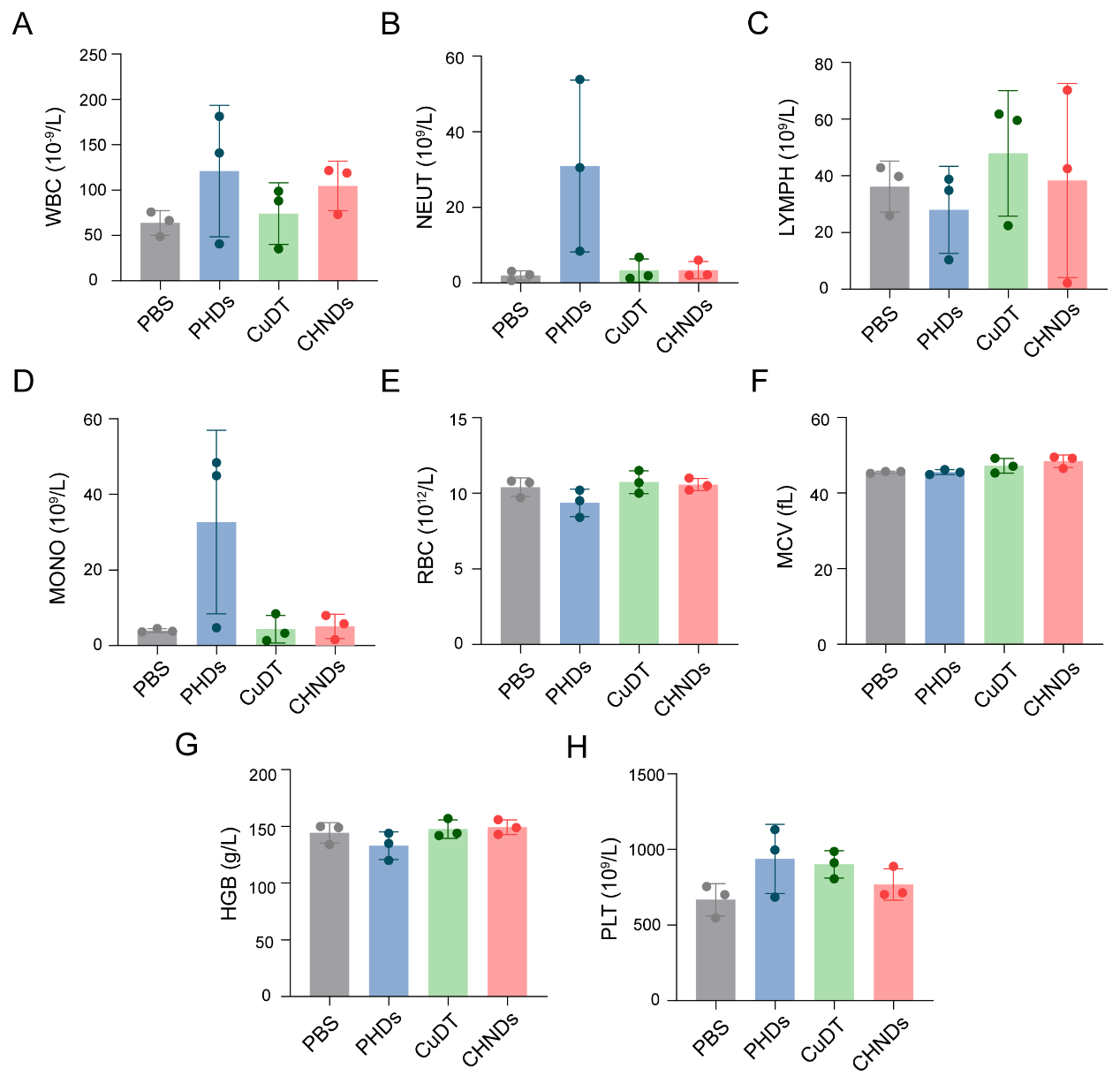


**Fig. S21.** Blood routine examination of 4T1 tumor bearing mice treated with indicated treatments.


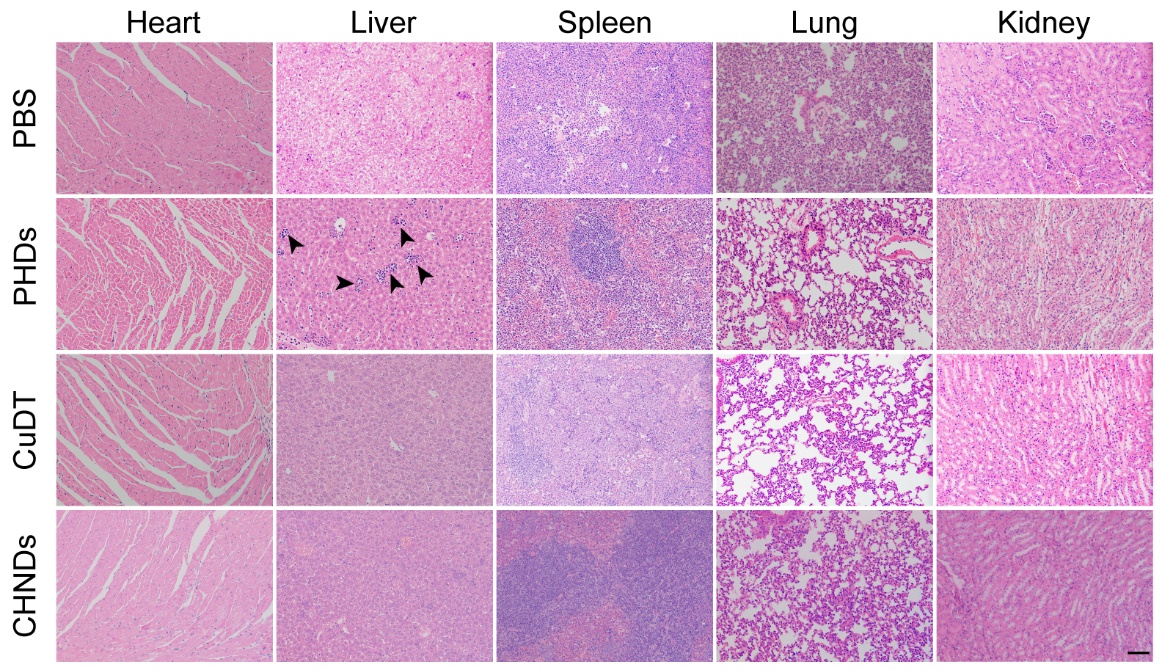


**Fig. S22.** H&E staining of major organs (heart, liver, spleen, lung, and kidney) in luciferase-expressed 4T1 tumor bearing mice after treated with different formulations (scale bar: 100 μm). The arrows indicated the region of liver inflammation upon treatment with PHDs.


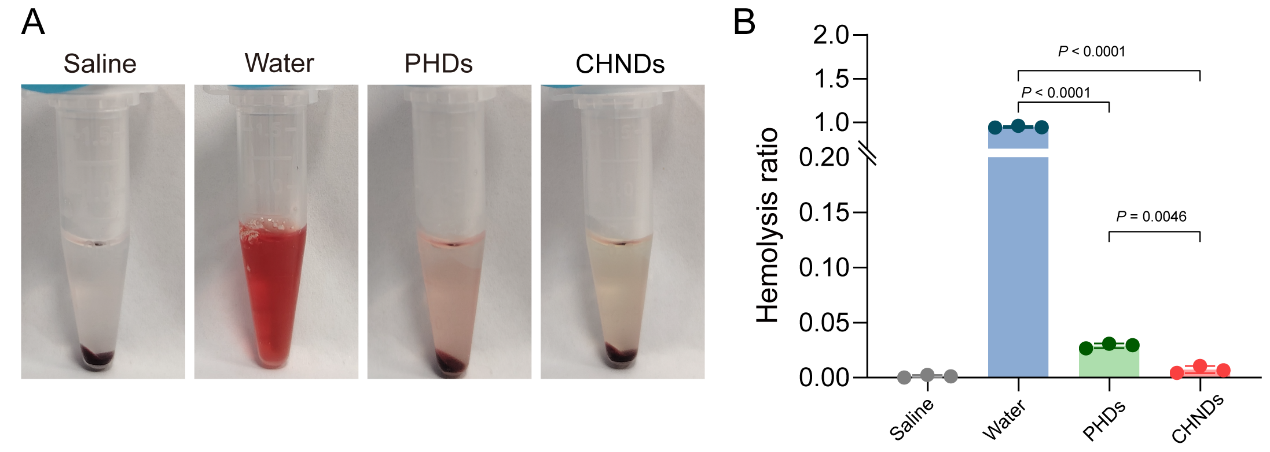


**Fig. S23.** *In vitro* hemolysis rate assay of CHNDs. **(A)** Representative images of hemolysis. **(B)** Hemolysis rates.


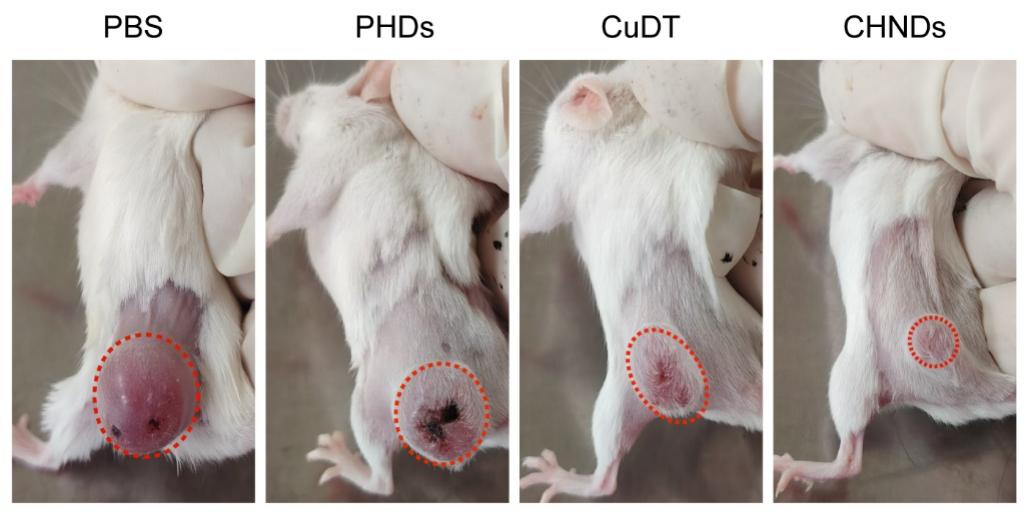


**Fig. S24.** Representative images of CT26 tumor bearing mice after treated with indicated treatments (2 mg 3BP/kg, 4.5 mg CuDT/kg, i.v.) for 5 times.


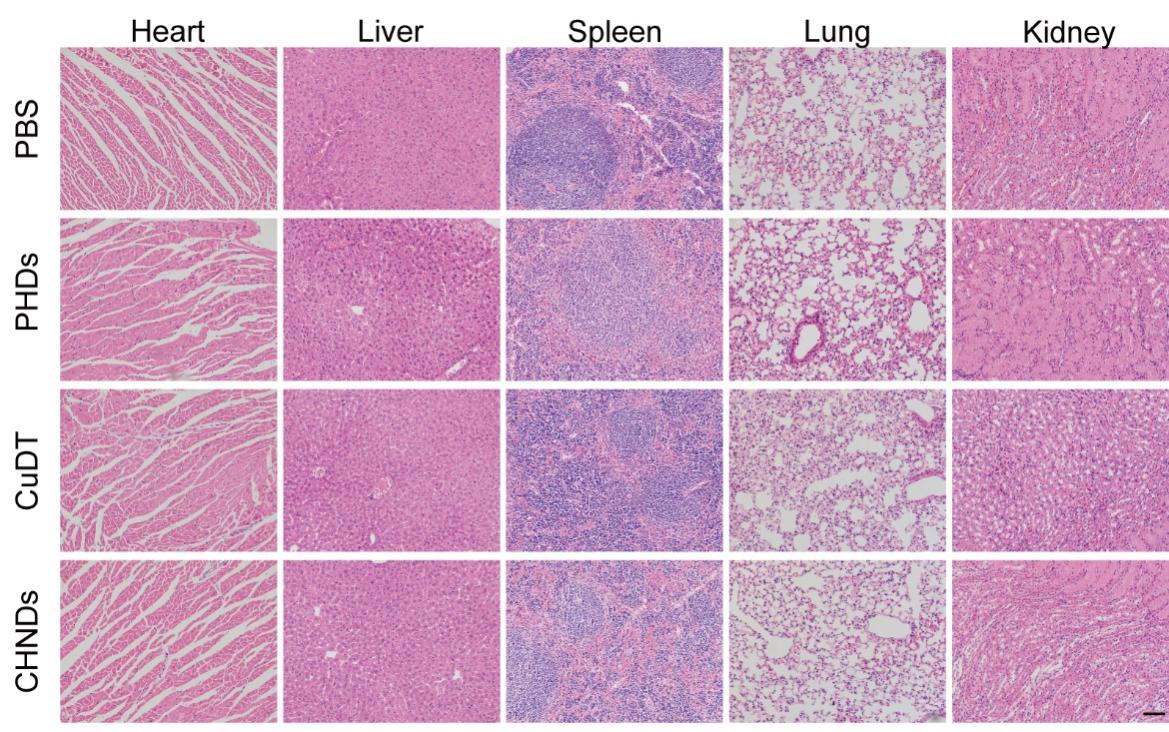


**Fig. S25.** H&E staining of major organs (heart, liver, spleen, lung, and kidney) in CT26 tumor bearing mice after treated with different drugs (Scale bar: 100 μm).
